# Reduced Self-Aggregation and Improved Stability of Silica-Coated Fe_3_O_4_/Ag SERS-Active Nanotags Functionalized With 2-Mercaptoethanesulfonate

**DOI:** 10.3389/fchem.2021.697595

**Published:** 2021-06-16

**Authors:** Maria Żygieło, Piotr Piotrowski, Marcin Witkowski, Grzegorz Cichowicz, Jacek Szczytko, Agata Królikowska

**Affiliations:** ^1^ Faculty of Chemistry, University of Warsaw, Warsaw, Poland; ^2^ Institute of Experimental Physics, Faculty of Physics, University of Warsaw, Warsaw, Poland

**Keywords:** surface-enhanced Raman scattering, plasmonic-magnetic nanocomposite, iron oxide nanoparticles, silver nanoparticles, SERS nanotags, magnetic self-aggregation, controlled clustering, silica coating

## Abstract

Nanocomposites combining magnetic and plasmonic properties are very attractive within the field of surface-enhanced Raman scattering (SERS) spectroscopy. Applications presented so far take advantage of not only the cooperation of both components but also synergy (enhanced properties), leading to multi-approach analysis. While many methods were proposed to synthesize such plasmonic-magnetic nanoparticles, the issue of their collective magnetic behavior, inducing irreversible self-aggregation, has not been addressed yet. Thus, here we present a simple and fast method to overcome this problem, employing 2-mercaptoethanesulfonate (MES) ions as both a SERS tag and primer molecules in the silica-coating process of the previously fabricated Fe_3_O_4_/Ag nanocomposite. The use of MES favored the formation of silica-coated nanomaterial comprised of well-dispersed small clusters of Fe_3_O_4_/Ag nanoparticles. Furthermore, adsorbed MES molecules provided a reliable SERS response, which was successfully detected after magnetic assembly of the Fe_3_O_4_/Ag@MES@SiO_2_ on the surface of the banknote. Improved chemical stability after coating with a silica layer was also found when the nanocomposite was exposed to suspension of yeast cells. This work reports on the application of 2-mercaptoethanesulfonate not only providing a photostable SERS signal due to a non-aromatic Raman reporter but also acting as a silica-coating primer and a factor responsible for a substantial reduction of the self-aggregation of the plasmonic-magnetic nanocomposite. Additionally, here obtained Fe_3_O_4_/Ag@MES@SiO_2_ SERS nanotags showed the potential as security labels for the authentication purposes, retaining its original SERS performance after deposition on the banknote.

## Introduction

Development of functional nanocomposites is a fast-growing branch of research in the advanced materials science. These nanocomposites can exhibit multiple or improved optical, electronic, magnetic, or catalytic properties compared to their individual single-component counterparts. Such multiphase structures were found useful in light harvesting (Liu et al., 2014; Xu et al., 2015), photoelectrochemistry (Kolodziejak et al., 2017; Kwiatkowski et al., 2017), nanomedicine (Inamuddin and Mohammad, 2018), sensing (Wang et al., 2012; Yuan et al., 2013), nanolasers (Passarelli et al., 2016; Galanzha et al., 2017), etc. In particular, combining plasmonic nanoparticles (NPs) with other nanostructured materials offers new properties and expands their functionality in chemical sensing. Among the techniques based on the surface plasmon resonance (SPR) phenomenon, surface-enhanced Raman scattering (SERS) spectroscopy is considered an exceptionally valuable analytical tool, offering the possibility of both sensitive and selective molecular analysis (Pilot et al., 2019; Langer et al., 2020). Nanocomposites exhibiting more than only plasmonic properties allow for the design of SERS-active materials showing multifunctionality (Han et al., 2013; Lai et al., 2013) and/or improved analytical performance: both in terms of limit of detection (LOD) (Du and Jing, 2011) and accuracy of the measurement (Frank et al., 2015).

**GRAPHICAL ABSTRACT:**
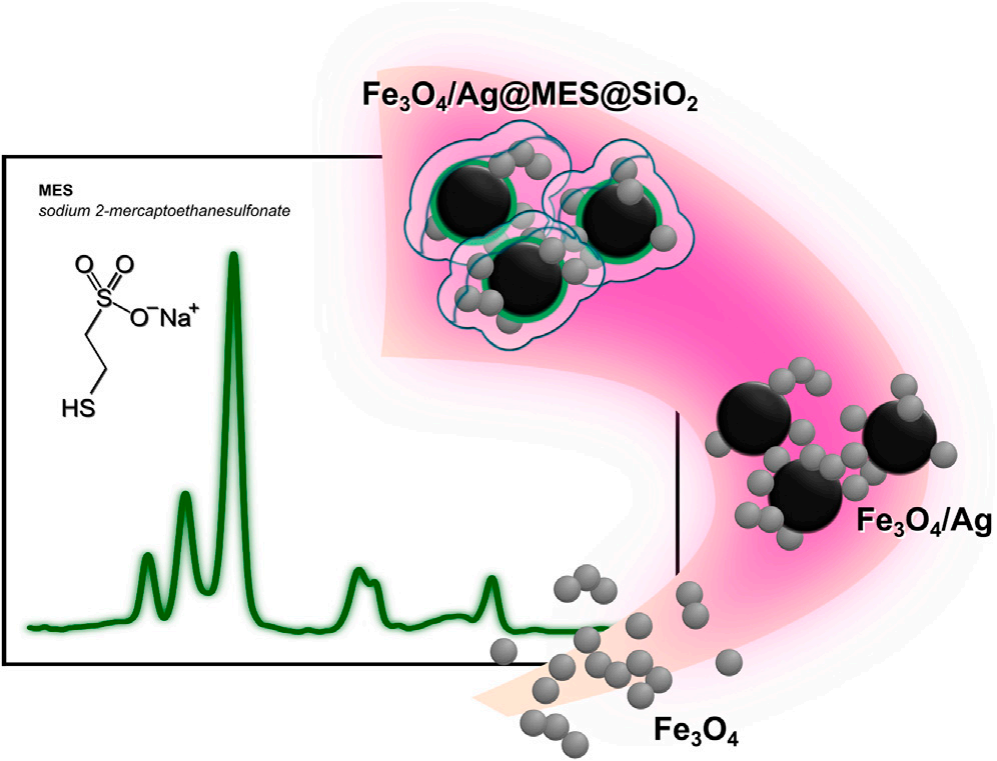


Various designs of multimaterial nanostructures were proposed to upgrade their applicability in SERS spectroscopy. For example, a metal-metal core-shell arrangement allows for meticulous control of the localized surface plasmon resonance (LSPR) wavelength by adjusting the geometry of the structure (Lu et al., 2010) and introduces, among others, the catalytic activity of the substrate, which provides a platform for monitoring of heterogeneous catalytic reactions with SERS spectroscopy (Bao et al., 2014; Yang et al., 2015; Zhang et al., 2020a). On the other hand, nanometal-nanocarbon conjugates act as efficient SERS substrates and are expected to merge optical and electronic properties (Fateixa et al., 2015; Jabłońska et al., 2018). Combination of graphene-related and plasmonic nanomaterials shows excellent SERS activity owing to the synergistic effect of the two components (Ding et al., 2013).

Among such multicomponent nanostructures, plasmonic-magnetic composites have gained particular interest. Although the synthesis of pure metallic nanoparticles is a relatively simple and low-cost method, poor control over these structures and limited formation of hot spots (regions of a very intense and highly localized electric field, e.g., within inter-particle junctions (Pilot et al., 2019) results in a strong discrepancy of the SERS signal detected with such systems. That is why aggregation controlled by an applied magnetic field is particularly important in SERS (Frank et al., 2015), since this results in generation of hot spots, overcoming two major hindrances crucial in SERS-based sensing: insufficient enhancement factor and irregular distribution of the functionalized nanoparticles across the sample.

Upon chemical modification, such a system acquires selectivity towards a chosen molecule or ion, useful in pollutant extraction and water purification (Pinheiro et al., 2018; Song et al., 2019; Fu et al., 2020). Apart from targeting a specific environmental contaminant, such composites are utilized in biochemistry for detection of DNA (Potluri et al., 2020) or cancer-related extracellular vesicles (Zhang et al., 2020b; Ning et al., 2020) as well as selection and sorting out cancer cells (Jun et al., 2010). Magnetically-controlled motion of SERS-active particles was also demonstrated to be helpful in designing the new-generation Raman barcodes for safety and security needs (Kim et al., 2010), on-demand targeting and enhanced SERS sensing, accomplished by remote manipulation (Wang et al., 2020) or constructing a multiplex immunoassay platform, based on combined SERS detection and magnetic capture (Neng et al., 2013).

Protective shells are often used for SERS-active nanomaterials to improve the long-term stability of colloidal NPs (Zhao et al., 2016) and to solve the issues related to desorption of the Raman active molecules or competitive adsorption of the interfering molecules (Li et al., 2018). Among the utilized shell materials, SiO_2_ is often the first choice as chemically inert (apart from high pH environments), transparent, hydrophilic, and bio-compatible (Li et al., 2017). As the prevalent method to encapsulate nanomaterials in silica, the modified Stöber reaction is employed (Stöber et al., 1968; Liz-Marzán et al., 1996). It involves hydrolysis and subsequent condensation of alkoxysilanes, such as tetraethylorthosilicate (TEOS), in alkaline water and alcohol mixture. This way, a dense silica layer is formed, permeable towards selected small objects such as metal ions (Piotrowski and Bukowska, 2016). Further modification of the procedure, i.e., templated growth with surfactants e.g., cetyltrimethylammonimum bromide or tetradecyltrimethylammonum bromide (Joo et al., 2009; Chen et al., 2013) results in a mesoporous shell with engineered porosity. As the surface of noble metal nanoparticles has low affinity to silica, there is a need to make it more vitreophilic. For this reason, metallic cores are functionalized with primer molecules. To date, various molecules have been used in this manner: NH_2_-functionalized silanes, such as (3-aminopropyl)-trimethoxysilane (APTMS) or (3-aminopropyl)triethoxysilane (APTES) (Liz-Marzán et al., 1996; Wong et al., 2011); polymers, both polyelectrolytes (PE), like poly(allylamine hydrochloride) (PAH) (Kim et al., 2010), and amphiphilic ones like poly(vinylpyrrolidone) (PVP) (Graf et al., 2003); and even simple organic anions, like citrates (Kobayashi et al., 2005). Ultimately, popular ionic SERS tags like *p*-mercaptobenzoic acid (pMBA) (Tian et al., 2016) or sodium 2-mercaptoethanesulfonate (Piotrowski and Bukowska, 2016) have been demonstrated to act as efficient primer molecules, facilitating the fabrication of SERS-active SiO_2_-coated nanosystems.

There are two distinctive approaches to SERS-based analysis with the use of silica-encapsulated plasmonic NPs. The first one involves deposition of the SiO_2_-coated SERS-active NPs directly on the probed surface. This method is called shell-isolated nanoparticle-enhanced Raman spectroscopy (SHINERS). It is based on the idea of forming a continuous dielectric shell, borrowing SERS enhancement from the plasmonic particle underneath, and bringing such a system into contact with the sample: the concept that was originally proposed by Li et al. (Li et al., 2010). Growing interest in SHINERS analysis over the last decade is related to the versatility of its use to surfaces of any composition and substrate morphology, as well as to the elimination of a direct influence of the metal structures on the examined molecules (Li et al., 2017; Krajczewski and Kudelski, 2019).

The second methodology relies on the adsorption of Raman active molecules inside the silica shell, directly on the surface of plasmonic particles (Küstner et al., 2009; Zhang et al., 2015). Systems fabricated using this approach can be further employed for the barcoding (Kim et al., 2010; Lai et al., 2015), construction of immunoassay (Ko et al., 2015; Neng et al., 2018) or bio-imaging based on the SERS response of such Raman labeled NPs (Rodríguez-Fernández et al., 2015).

Coating plasmonic NPs with a thin layer of silica is beneficial to SERS spectroscopy for numerous reasons. The silica layer not only improves the chemical stability of the NPs and provides the ability to disperse them in water easily, but also protects the Raman probe, reduces the undesired interactions, and facilitates surface modification toward further development of the system (Li et al., 2017). Additionally, due to the transparency of the SiO_2_ material, both the plasmon-enhanced incident laser beam and the scattered radiation can penetrate the silica layer, around the metallic NP. All these features boost SERS performance and increase the SERS-related applicability of the carefully designed silica-coated plasmonic nanomaterials. Protecting role of the deposited SiO_2_ layer was demonstrated for the decahedral AgNPs exposed to yeast cells, which prevented decay of the SERS signal, often occurring very fast for bare AgNPs (Koła̧taj et al., 2017). Another interesting use of silica shell-modified SERS-active NPs is pH sensors, including intracellular applications, with pMBA as the most exploited Raman reporter (Kneipp et al., 2007; Jaworska et al., 2015). SERS response of such a sensor can be disturbed in a real-life biological system by the contact of Raman reporter molecules with the proteins present in the system, influencing the dissociation of the pMBA carboxylic group, and thus distorting the SERS readout of pH of the local environment. Wang et al. (Wang et al., 2012) showed that pMBA-functionalized AgNPs coated with a 30 nm thick SiO_2_ layer with small pores prevented bovine serum albumin (BSA) molecules from interacting with the reporter molecule, preserving the pH sensitivity of the system. Silica-encapsulated dimers of AgNPs, functionalized with pMBA molecules as SERS tags and further covalently conjugated with the antibodies as targeting ligands were successfully used for imaging cancer cells (Xia et al., 2013). Good specificity and high sensitivity were both achieved with this method, proving the flexibility of SiO_2_ layer design to inhibit the nonspecific binding. Moreover, superior SERS enhancement of the spherical silver dimers to single Ag nanospheres and nanocubes with similar sizes was demonstrated for the described system (Xia et al., 2013). This effect is related to the generation of hot spots due to the plasmon coupling within the interparticle gap (Pilot et al., 2019). Interestingly, it was also confirmed that monomeric Ag and AuNPs are SERS inactive, which was nicely illustrated using the colloidal suspensions of silica-encapsulated NPs (Zhang et al., 2014). Thus, the dimers, trimers, or larger clusters of NPs are necessary to form the hot spots, providing satisfactory sensitivity for the SERS experiment. Use of the dimers with a well-defined size of hot-spot gap ensures good reproducibility of the SERS signal (Wustholz et al., 2010; Xia et al., 2013), however even partial control over aggregation of NPs is advantageous.

Preventing the contribution of random aggregation is particularly challenging for magnetic NPs. Iron oxide NPs are known to form reversible agglomerates or even permanent aggregates in a liquid phase, facilitated by spontaneous magnetic attraction (Gutiérrez et al., 2019). This issue becomes even more critical when aiming to re-disperse agglomerated powder. Even though spontaneous agglomeration is favorable from the point of SERS intensity, it may hinder further engineering of the system. Presence of the aggregates has a strong impact on the magnetic properties of such colloidal suspension, as well as it may hamper subsequent surface modification and/or attachment of next components to the system. Therefore, special efforts have been made to control the aggregation of magnetic NPs. The most common strategies are the use of peptizers (Lu et al., 2006) or ultrasonic treatment (Yang et al., 2005), which were successfully used for preparation of Fe_3_O_4_/SiO_2_ nanocomposites.

Herein, Fe_3_O_4_/Ag nanocomposite synthesized by *in situ* reduction of the silver ions was used as a platform onto which SiO_2_ layer was formed, using a sulfonate-terminated thiol, 2-mercaptoethanesulfonate ions (MES), as a primer molecule for the first time in such a complex system. For the nanomaterial presented here, we struggled with a spontaneous magnetic attraction between Fe_3_O_4_ NPs at two steps of the synthesis: deposition of AgNPs and subsequent silica coating of the Fe_3_O_4_/Ag nanocomposite. While the sonication of the magnetite NPs dispersion at the first stage was satisfactory to avoid the uncontrolled aggregation, it was insufficient in the case of encapsulating Ag-deposited magnetite NPs with SiO_2_ (to form Fe_3_O_4_/Ag@SiO_2_).

In this work, we propose a straightforward strategy for fabrication of the SERS-active plasmonic and magnetic nanocomposite, using MES as both the Raman tagging molecule (SERS marker) and the priming agent that facilitates grafting the SiO_2_ layer on the Fe_3_O_4_/Ag nanocomposite surface. The controlled clustering of Fe_3_O_4_/Ag particles occurred preferentially in the presence of MES molecules during the silica coating process. The SiO_2_ layer additionally minimizes agglomeration of the final nanocomposite, maintaining the reduced but still reliable enhancement of SERS signal due to MES molecules, compared to bare Fe_3_O_4_/Ag nanocomposite. Assembly of the Fe_3_O_4_/Ag and Fe_3_O_4_/Ag@SiO_2_ particles with the external magnetic field resulted in the improved density of hot spots (per unit area of the sample), while the absolute SERS intensity was nearly not influenced by the presence of the magnet when silica coating was applied.

Presence of the SiO_2_ layer also promoted the improved stability of the material against dissolution upon yeast cells. Eventually, successful application of the here designed Fe_3_O_4_/Ag@MES@SiO_2_ nanomaterial as SERS-tags was demonstrated, exhibiting superior properties to its free-of-silica-shell counterpart. Consequently, here presented SiO_2_-protected, Raman-tagged Fe_3_O_4_/Ag nanocomposite is expected to bring an advantage in analysis and labeling by means of SERS spectroscopy and other fields where nanomaterials that combine plasmonic and magnetic properties can be readily applied.

## Materials and Methods

### Chemicals

Ferric chloride hexahydrate (FeCl_3_·6H_2_O, 97.0%), ferrous chloride tetrahydrate (FeCl_2_·4H_2_O, 99%), poly(allylamine hydrochloride) (PAH, MW ∼450,000 Da), poly(acrylic acid) (PAA, MW ∼50,000 Da), *n*-butylamine (*n*-ButNH_2_, 99.5%) and tetraethoxysilane (TEOS, 98.0%) were purchased from Sigma-Aldrich Chemistry. Silver nitrate (AgNO_3_, >90.0%, Honeywell) and sodium 2-mercaptoethanesulfonate (MESNa, >98.0%) were supplied by Fluka. Ammonia solution (NH_3_·H_2_O, 25.0%) was bought from Chempur. Ethanol (99.8% and 96.0%), hydrochloric acid (35.0–38.0%) and nitric acid (65.0%) were acquired from POCH S.A. All chemicals were used as supplied, without further purification. Lyophilized *Saccharomyces boulardii* cells from Enterol 250 (BIOCODEX) capsules (probiotic yeast) were also used.

All glassware was treated with freshly prepared aqua regia (HCl:HNO_3_=3:1; v:v), followed by rinsing with copious amounts of Milli-Q water prior to further use. Ultrapure water (Millipore Milli-Q system, 18.2 MΩ·cm) was used to prepare all aqueous solutions. Nitrogen gas (≥99.999%) used for removal of dissolved oxygen was provided by Air Products.

### Methods of Synthesis

#### Synthesis of Fe_3_O_4_ Nanoparticles

Magnetic nanostructures of mixed iron oxide were obtained by co-precipitation technique, using a mixture of iron salts as precursors with a strictly defined stoichiometry, under the influence of precipitating agent in the alkaline medium. The reaction was performed based on the procedure described in (Yu et al., 2014), while historically the first controlled synthesis of superparamagnetic iron oxide particles using alkaline precipitation of FeCl_2_ and FeCl_3_ was introduced by Massart (Massart, 1981).

Reaction precursors: ferric chloride and ferrous chloride were dissolved in 2:1 molar ratio (4.88 and 2.99 g, respectively) in 150 ml of water under the nitrogen flow. Then, 26% ammonia solution was added dropwise under mechanical stirring, until the pH value reached 10.2. Next, the mixture was stirred vigorously for 10 min and then it was heated up to 85°C for 30 min. Black Fe_3_O_4_ precipitate was separated from the supernatant by a permanent magnet and rinsed under the magnetic field with water and ethanol, 3 times each. The final product was dried in the vacuum oven in 65–70°C for 2 or 3 h and was used in the further experimental step in the powder form.

#### Synthesis of Ag-Decorated Fe_3_O_4_ Nanoparticles (Fe_3_O_4_/Ag)

The Fe_3_O_4_/Ag nanocomposite was obtained by *in situ* reduction of silver ions with a reducing agent, according to the modified procedure by Kim et al. (Kim et al., 2010). In order to design a nanomaterial with good SERS performance, different amounts of reagents were examined to optimize the conditions for the reaction of AgNPs deposition. Previously dried Fe_3_O_4_ NPs were re-dispersed (1 mg/5 ml or 2 mg/5 ml) in AgNO_3_ solution (2, 4, 8 and 16 mM) in ethanol. The mixture was sonicated for 30–45 min before adding neat *n*-ButNH_2_ in a various molar ratio with respect to AgNO_3_ (1:1, 1:5).

The following conditions generate the composite with the best SERS activity: 2 mg/5 ml of Fe_3_O_4_ NPs, 8 mM AgNO_3_ and 1:1 Ag^+^ to *n*-ButNH_2_ molar ratio. In brief, 24.7 mg of Fe_3_O_4_ NPs was added to 60 ml of ethanol containing 82.2 mg AgNO_3_; then the mixture was sonicated for 30–45 min before adding 47.2 µl of neat *n*-ButNH_2_. The reaction was performed in a polypropylene container under continuous shaking for 50 min at 50°C. The final suspension of Fe_3_O_4_/Ag was cooled to room temperature and rinsed 2 times with ethanol under the external magnetic field. Separated solid precipitate was dried under vacuum at 65 C for approximately 1.5–2.0 h.

#### Adsorption of MES Molecules (Raman Marker)

Adsorption of MES molecules on Fe_3_O_4_/Ag surface was performed by suspending solid Fe_3_O_4_/Ag (brown powder) in a concentration of 2 mg/ml in 1 mM MES solution (typical reaction volume was around 2 ml). The Fe_3_O_4_/Ag@MES suspension was first sonicated for about 30 min prior to the stationary phase of adsorption, proceeding from 1 to 12 h. After that time, the Fe_3_O_4_/Ag@MES precipitate was separated from the supernatant under the magnetic field, washed two times with water, and re-dispersed in water up to the initial volume.

#### Silica Encapsulation of Magnetic-Plasmonic Nanocomposites

The fabrication of the SiO_2_ shell was carried out using a modified Stöber procedure (Kim et al., 2010). The catalytic reaction (TEOS hydrolysis and condensation) was initiated by adding 50 µl of 9.4 M aqueous ammonia solution and 50 µl of TEOS to the suspension containing about 2 mg of Fe_3_O_4_/Ag@MES@PE or Fe_3_O_4_/Ag@MES in 3.5 ml of ethanol and 0.5 ml water. The freshly prepared MES-functionalized Fe_3_O_4_/Ag nanocomposite (bare or with polyelectrolyte layer) was introduced in a form of a slightly wet slurry (the supernatant from the previous reaction step was discarded). The reaction mixture was left under continuous sonication for about 15–25 min. Next, it was transferred onto a laboratory shaker and left for 2 h at room temperature. The final product was sonicated, then magnetically separated and purified in two ways: either by rinsing three times with water (3xW) or consecutively with water, ethanol and once again water (WEW).

#### Coating of Fe_3_O_4_/Ag@MES With Polyelectrolyte Layers (Fe_3_O_4_/Ag@MES@PE)

As an alternative strategy for the direct formation of a SiO_2_ layer with MES as primer molecules, described above, the nanocomposite with adsorbed MES molecules was coated with polyelectrolyte (PE) films prior to the formation of a silica layer (Kim et al., 2008). Fe_3_O_4_/Ag@MES in a form of wet precipitate (2.0 mg, after discarding the supernatant) was immersed seven times in 1 ml of polyelectrolyte solution: alternately in 1 mg/ml solution of PAH and 1 mg/ml solution of PAA (starting with PAH). This way, the coating consisted of casting 4 layers of PAH and 3 layers of PAA. Initial layer of PAH was necessary to ensure electrostatic attraction of negatively charged MES molecules adsorbed onto Fe_3_O_4_/Ag nanocomposites surface. For each layer the whole suspension was first sonicated for a few minutes, then it was left for 10 min (at room temperature) and rinsed with 1 ml of water under the magnetic field before changing the polyelectrolyte type. Subsequently, the outermost PAH layer was subjected to the silica encapsulation process, following the procedure already described in *Silica Encapsulation of Magnetic-Plasmonic Nanocomposites*.

#### Chemical Stability of Nanocomposite Toward Yeast Exposure

The samples for testing the stability of a given nanocomposite were prepared by mixing the room-temperature suspension of yeast cells in ultrapure water (100 ml suspension containing roughly 2.5 mg of yeast cells per ml was mixed manually for a few minutes, left stationary for 30 min and next decanted, using an upper part of the supernatant for further studies) with a fridge-stored ethanolic colloidal suspension of nanomaterial in 1:1 (v/v) ratio (1 ml of both yeast and NPs).

Fe_3_O_4_/Ag@MES@SiO_2_ was examined, while the Fe_3_O_4_/Ag@MES was employed as a reference sample. To this end, nanocomposite obtained respectively before or after the SiO_2_ encapsulation was diluted 10 times with water and sonicated for a few minutes before mixing with yeast. The samples after 1 or 48 h exposure to yeast solution were investigated.

### Characterization Techniques

#### PXRD Measurements

Powder X-ray diffraction (PXRD) patterns were collected on a Bruker D8 Discover diffractometer with Debye-Scherrer geometry. All samples were dried, ground, and sealed in glass capillaries (diameter 0.5 mm, Hampton Research). For all three samples, measurement procedure included 15 repeated scans, and as there were no changes in acquired diffraction patterns, collected data for each sample were added.

Every single scan was performed using Cu Kα radiation (λ = 1.540598 Å), at a scan rate of 0.72°/min in 0.012° steps, covering the range of 2θ from 20° to 130°. All obtained data were analyzed with the aid of DIFFRAC.EVA and TOPAS 4.2 software.

#### (S)TEM and EDX Imaging

##### (S)TEM and EXD Instrumentation and Sample Preparation

Transmission electron microscopy (TEM) studies were performed using Zeiss LIBRA 120 TEM microscope (Zeiss, Germany), operating at an accelerating voltage of 120 kV. Formvar-coated 300-mesh copper grids were used to prepare samples for TEM analysis. A drop of nanomaterial suspension was placed on the grid and left to dry under ambient conditions.

Additional investigations in scanning transmission electron microscopy (STEM) mode were conducted on an FEI Talos F200X transmission microscope at 200 kV. The measurements were performed using a high-angle annular dark-field (HAADF) detector. Energy-dispersive X-ray spectroscopy (EDX) on a Brucker BD4 instrument was employed for mapping atomic distribution. The samples for the STEM observations were prepared by dropping the colloidal particles suspension on amorphous carbon film supported on a 300-mesh copper grid and left for solvent evaporation.

##### (S)TEM Images Processing

Feret’s diameter, defined as the longest distance between two points placed on the outline of the selected object, was calculated from the TEM images using the ImageJ software (Wayne Rasband, National Institutes of Health (NIH), Bethesda, MD, Unites States), running in Java. Histograms showing the nanoparticle size distribution and corresponding Gaussian fit of the data were plotted, using OriginPro 2019 software.

#### SQUID Magnetometry

##### Magnetic Properties

Magnetic properties of nanoparticles were investigated using the Quantum Design MPMS-7 SQUID (superconducting quantum interference device) magnetometer. Solid samples were encapsulated in a Parafilm^®^ M envelope, whose mass was determined before and after the implantation of the sample using Sartorius SE2 ultramicrobalance.

Magnetization of samples was determined at 300.0 and 2.0 K as a function of external magnetic field up to 70,000 Oe (7.0 T). Hysteresis loops were measured by reversing the direction of the magnetic field down to −12,000 Oe (1.2 T).

Diamagnetic contribution from the capsule, estimated by scaling the magnetization measured for a neat Parafilm^®^ M sample, was subtracted during data post-processing for all measurements.

Saturation magnetization was estimated using two methods: by choosing the highest measured magnetization and by fitting the Langevin function (McElfresh, 1994; Cullity and Graham, 2008; Ozkaya et al., 2009):

$$M=M_{s}\left(\coth\left(\frac{\mu H}{k_{B}T}\right)\;-\frac{k_{B}T}{\mu H}\right)$$ (1)

where: M is the measured magnetization, $M_{s}$ is the saturation magnetization (fitting parameter), H is the magnetic field strength, $k_{B}$ is the Boltzmann constant [$1.3806505\left(24\right)\cdot 10^{-23}\frac{J}{K}$ (Mohr et al., 2005)], T is the temperature of the measurement and μ is the mean total magnetic moment of a single particle, which was the second fitting parameter.

Fitting was performed using the NonlinearModelFit procedure in the Wolfram Language, executed with Wolfram Mathematica 10.3 software.

For the detailed discussion on the measurement uncertainties in SQUID analysis, the reader is referred to the Chapter 1.1 of the Supplementary Material.

Temperature-dependent measurements of magnetization were also performed and the precise details of the experiment are presented in the Chapter 1.2 of the Supplementary Material.

##### Mass Percent Composition

The mass contents of Fe_3_O_4_, Ag, MES, and SiO_2_ were determined using the relative differences in the highest observed saturation magnetization for the nanomaterial obtained at various steps of the synthesis. A few assumptions were made in order to estimate the mass composition:

1. Diamagnetic contribution of silver, MES, and silica is negligible in comparison to the magnetization of Fe_3_O_4_.
2. Consecutive steps of synthesis consist only of the addition of an individual component and do not introduce any contaminations.
3. All surface modifications do not change the magnetic properties of Fe_3_O_4_.
4. The relative mass concentration of existing components is not affected by the appearance of a new one (i.e., mass ratio of Ag and Fe_3_O_4_ is the same for all samples containing these two components).
5. The measurement uncertainties are independent.

Using the abovementioned assumptions one is able to calculate the relative mass percent of a component introduced in a particular step of the synthesis by calculating the dilution of magnetization with a comparatively non-magnetic component.

#### Normal Raman and SERS Spectroscopy

##### Raman Spectroscopy Instrumentation

Normal Raman (NR) and SERS spectra were measured on a dispersive Labram HR800 (Horiba JobinYvon) spectrometer, coupled with Olympus BX61 confocal microscope and Peltier-cooled charge-coupled device detector. In most of the experiments, a built-in He-Ne laser was used (632.8 nm) with a 50x objective lens and a holographic grating with 600 grooves/mm. Alternatively, 532 nm frequency-doubled Nd:YAG laser was applied as an excitation source. The full power of a laser beam at the sample was around 3 mW, further reduced 4 or 10 times using the neutral density filter, if necessary. Spectra were collected in a back-scattering configuration. Calibration of the system was performed with respect to the 520 cm^−1^ band of a silicon wafer.

##### Sample Preparation and Data Collection

Samples were deposited on the surface of the glass microscopic slide and left for solvent evaporation under ambient conditions. Each colloidal suspension was first sonicated for 15–30 min, prior to further sample preparation. In the case of the stability test, a magnet-assisted collection of the nanocomposite was employed already for the mixture of the nanocomposite yeast cells for the silica-coated nanomaterial.

A neodymium magnet was placed in proximity to the slide and the nanomaterial was carefully aggregated in case of the samples prepared under the external magnetic field unless stated otherwise when presenting the results. The samples were typically dried under ambient conditions, while heating at 38–40°C for 10 min was applied in case of the experiment with yeast cells.

The typical NR spectrum was an average of 2 accumulations, 60 s each. For SERS spectra the acquisition time for a single accumulation varied from 10 to 30 s, while the number of the accumulations was 3 for each spectrum. Single point NR and SERS spectra were acquired typically in a range of 200–1800 cm^−1^.

Point spectral Raman mapping was carried out for the needs of statistical analysis of SERS performance of prepared nanomaterials. SERS signal from 20 to 36 different spots across the sample was typically acquired for each map. In this manner, the reproducibility of the SERS intensity was evaluated for the signal recorded from different areas of the same sample and between various samples prepared using the same method and/or under changing experimental conditions.

##### SERS Data Analysis

SERS spectra were further analyzed after initial baseline correction in LabSpec 5. VBA macro executed in Microsoft Excel facilitated the extraction of single spectra from the maps, useful in determining the average (arithmetic mean) maximum intensity of the selected MES marker band and its standard deviation (SD). This approach was successfully validated several times for random data sets, by comparing the result with the analogous parameter, calculated using the average (arithmetic mean) spectrum generated by VBA macro and obtained manually using software features (peak fitting tool) implemented in LabSpec 5. When necessary, small shifts of the wavenumber corresponding to the maximum intensity of the MES band were included in the procedure for determining the average intensity value. The accumulation time varied between the experimental series, but all the values of SERS intensity are presented in counts per second [cps], to enable their direct comparison.

##### Normalization Procedure of SERS Intensity

In order to minimize the effect of temporal laser power fluctuations in the used Raman set-up on the collected SERS spectra, we performed intensity normalization with respect to the Si signal. Normalization process required systematic recording of Raman signal for the silicon wafer over time with the same experimental parameters, i.e.: 632.8 nm excitation beam, 50x objective lens, and no use of laser beam filter attenuator.

Normalization of SERS intensity results (from various sample areas, collected in the mapping mode) involved four main steps: analysis of raw silicon wafer data in LabSpec 5 software (including baseline cut-off and careful read-out of the maximum band intensity), calculation of normalization factor (*NF*), by dividing silicon wafer signal in the particular day of the experiment $\left(I_{Si}\right)$ by its maximum signal value among all the recorded signals within the period when the described studies were carried out $\left(I_{Si}^{max}\right)$:

$$NF=\frac{I_{Si}}{I_{Si}^{max}}$$ (2)

and, finally, normalization of the SERS signal collected from the composite in the following manner. In order to get normalized values ($I_{SERS}^{norm}$) , the average intensity of the SERS signal derived from the mapping experiments done on a particular day (of a given nanomaterial sample) was divided by the value of *NF* from the same date:

$$I_{SERS}^{norm}=\frac{\langle I_{SERS}\rangle }{NF}$$ (3)

The same procedure was applied to normalize the mean signal of SERS-active nanocomposite samples for bare and silica coated Fe_3_O_4_/Ag@MES nanocomposites.

The last step was to take into account the type of a laser attenuator. We accomplished it by dividing the ($I_{SERS}^{norm}$) [cps] by the laser beam power reduction factor. All SERS results are presented for the same filter, reducing the beam power by a factor of 10.

Summarizing, all SERS intensities presented in the manuscript were normalized according to the procedure described above.

#### UV-Vis Spectroscopy

Extinction spectra were collected using a Thermo Scientific Evolution 201 UV-Vis spectrophotometer. In case of mixtures with yeast cells, nanocomposite was separated and re-dispersed in a fresh portion of water before measurements .

## Results and Discussion

### Synthesis and Properties of Fe_3_O_4_/Ag@MES@SiO_2_ Nanocomposite

In this study we focused on the fabrication of SERS-active and magneto-responsive Fe_3_O_4_/Ag nanocomposite, coated with a relatively thin layer of silica, as well as on demonstration of the applicability of the material as nanotags. However, in this section, we describe not only the method of synthesis and properties of the final best-performance product but also briefly present the key steps of optimization during the synthetic route design.

#### Operating Principle

This work aimed at addressing the issue of self-aggregation of silica-coated magnetic-plasmonic nanocomposites on the example of hybrid nanostructures composed of plasmonic AgNPs attached to a magnetic Fe_3_O_4_ core, embedded in a SiO_2_ shell. Combining magnetic and plasmonic properties is of a general high interest, as it improves SERS performance and paves the way for new fascinating SERS-based applications, compared to pure silver nanoparticles. Coating of the Fe_3_O_4_/Ag nanostructures with a silica layer was performed, in order to extend the stability of the whole system. Molecules of 2-mercaptoethanesulfonate (MES) were used as a SERS marker, whose main advantage, apart from being a strong Raman scatterer (Kudelski, 2002), is its photostability, which is often a key issue to a successful SERS performance (Huang et al., 2010). MES molecules played a twofold role: a SERS tag and a priming agent for the silica layer growth.

**FIGURE 1:**
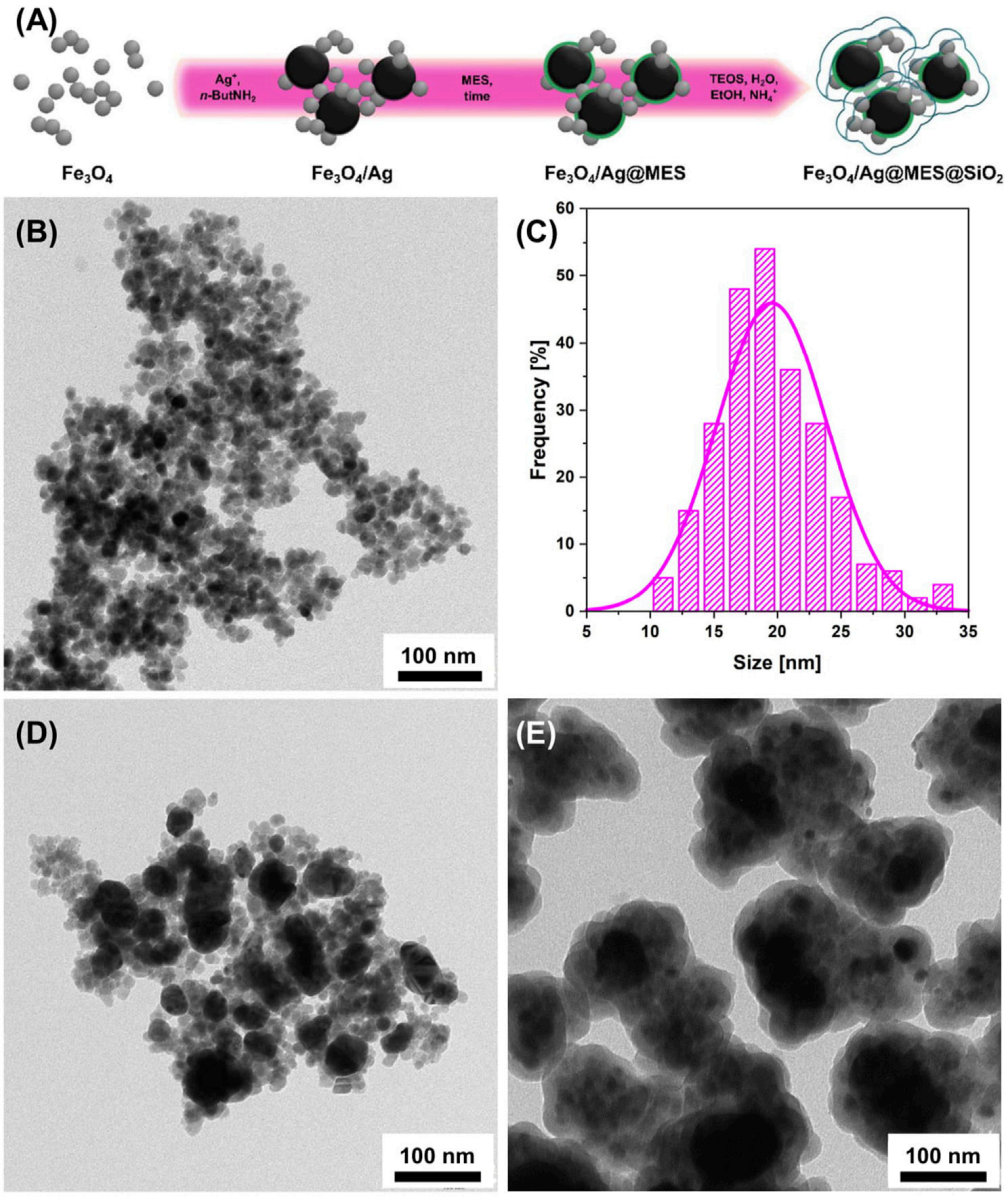
**(A)** Schematic illustration of the preparation of Fe_3_O_4_/Ag@MES@SiO_2_ nanocomposite. Representative TEM images of **(B)** Fe_3_O_4_ (together with histogram showing the particle size distribution of this sample in part **(C)**; **(D)** Fe_3_O_4_/Ag and **(E)**. Fe_3_O_4_/Ag@MES@SiO_2_ nanomaterials.

The scheme of the fabrication process of Fe_3_O_4_/Ag@MES@SiO_2_ is illustrated in Figure 1A. Fe_3_O_4_ NPs are smaller and shown in grey, while the larger, black particles correspond to AgNPs (to reflect the size and contrast difference observed in the TEM images). First, Fe_3_O_4_

NPs were prepared using the coprecipitation technique (Yu et al., 2014). Next, AgNPs obtained by *in situ* reduction of silver ions with *n*-ButNH_2_ were attached to the magnetic core. The adsorption step of MES molecules involved 12 h of self-assembly. Finally, the adsorbed MES layer was protected with silica coating fabricated in a modified Stöber reaction (Kim et al., 2010). Each step of the synthesis was followed by a magnetic field-assisted separation and further purification of the nanomaterial. The representative TEM images of the nanomaterial prepared at each stage of the synthesis (except MES adsorption) are shown in Figures 1B,D,E. The fabricated Fe_3_O_4_ NPs were primarily of spherical shape (see Figure 1B), while the mean particle size deduced from TEM imaging was about 19.6 ± 4.3 nm in Feret’s diameter (see the histogram plot in Figure 1C). AgNPs were successfully formed on the surface of the previously synthesized Fe_3_O_4_, as evidenced by the dark and larger objects distributed on the brighter and smaller ones, visible in TEM image shown in Figure 1D. The silver nanoparticles remained attached to the Fe_3_O_4_ NPs after several washing steps, which suggests some interactions between the magnetic and plasmonic components. The average size of the silver nanoparticles determined from the TEM analysis was 72.5 ± 32.7 nm. In the final step, controlled clustering of Fe_3_O_4_/Ag particles was observed, occurring preferentially in the presence of MES adsorbate prior to silica coating process (see Figure 1E). Similar performance of MES anions, namely protection from nanoparticles aggregation, preventing dissolution of silver in ammonia (during Stöber reaction) and acting as a primer in the silica-coating process has been already reported for the hydroxylamine reduced AgNPs (Piotrowski and Bukowska, 2016), for which shell formation was demonstrated for individual silver particles.

The resulting Fe_3_O_4_/Ag@MES@SiO_2_ nanocomposite that exhibited the best performance acted as a multifunctional nanomaterial; that was not only easily separated from the suspension and deposited on a solid substrate via the magnetic controlled-assembly, but also served as effective and stable SERS nanotags. Application of external magnetic field enables facile collection of the nanomaterial and formation of a uniform and dense layer of the nanocomposite on the surface. Such prepared layer provides a large number of dense and homogenously arranged hot spots that are expected to present a high value of SERS enhancement. Thus, the intense and reproducible SERS signal of MES marker could be readily obtained. Protective SiO_2_ encapsulation is a trade-off between the SERS efficiency and stability of the nanotags.

#### PXRD, (S)TEM, and SQUID Characterization of the Best-Performance Product

**FIGURE 2:**
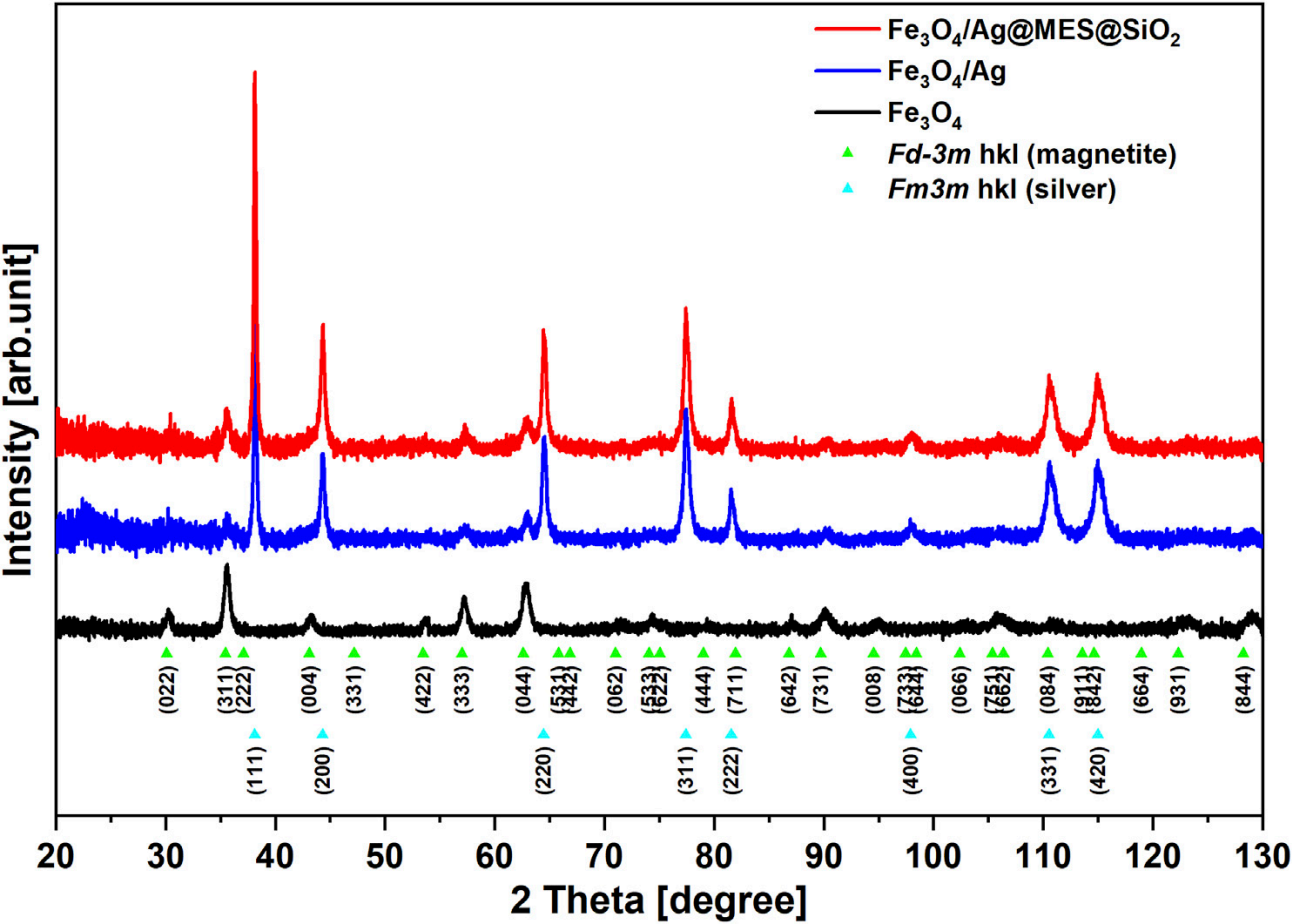
PXRD patterns of the products synthesized at given preparation step (see legend). Patterns were indexed to *Fd−3m* magnetite and *Fm3m* silver groups.

Phase analysis for all the above synthesized products at each preparation step (except MES adsorption) was performed using X-ray powder diffraction (PXRD). Figure 2 shows comparison of diffraction patterns for samples of Fe_3_O_4_ nanoparticles synthesized via coprecipitation (black curve), AgNPs decorated magnetic nanocomposite of Fe_3_O_4_/Ag (blue curve) and silica coated Fe_3_O_4_/Ag@MES@SiO_2_ nanocomposite (red curve). The diffraction pattern of Fe_3_O_4_ nanoparticles exhibits typical set of reflection characteristic for magnetite (Fe_3_O_4_) crystalline phase (*Fd*−3*m* space group) (Fleet, 1981). The strongest and most distinguishable reflection occurs at the value of 2θ = 35.6° and is corresponding with the (311) planes of cubic symmetry lattice. Similar set of reflections can also be found in the diffraction patterns of other samples, thus the obtained magnetite nanoparticles were stable during subsequent modification with silver and silica. In diffraction patterns of Fe_3_O_4_/Ag and Fe_3_O_4_/Ag@MES@SiO_2_ (see blue and red curves in Figure 2) second crystalline phase is also visible. Sharper and more intensive reflections with 2θ values of 38.2°, 44.3°, 64.5° and 77.4° are associated with a cubic lattice symmetry of silver nanoparticles (*Fm*−3*m* space group) (Wyckoff, 1963). In the diffraction pattern of Fe_3_O_4_/Ag@MES@SiO_2_ nanocomposite there is no reflection which can be associated with SiO_2_ phase, so it could indicate that silica occurs only in an amorphous form. Pawley fitting procedure was performed for the all crystalline phases.

Calculated values of lattice constant (*a*) are shown in Table 1. Moreover, the average crystallite size (*d*, calculated as volume-weighted mean crystallite size (Balzar et al., 2004)) was estimated for all crystalline phases, being in a range of about 15 and 50 nm for the magnetite and silver, respectively (see Table 1 for the exact values). These values for Fe_3_O_4_ are smaller than those determined from TEM images (see *Operating Principle*), but this discrepancy can be simply related to the fact that particle size (probed by TEM) may consist of multiple crystallites (average crystallite size is obtained from PXRD).

**TABLE 1:** Lattice constants and average crystallite size of magnetite and silver nanoparticles phases.

Sample	*a*(Fe_3_O_4_) [Å]	*a*(Ag) [Å]	*d*(Fe_3_O_4_) [nm]	*d*(Ag) [nm]
Fe_3_O_4_	8.366	−	14.3	−
Fe_3_O_4_/Ag	8.362	4.085	13.2	48.1
Fe_3_O_4_/Ag@MES@SiO_2_	8.367	4.086	15.6	53.8

As can be noticed in Figure 2, the diffraction peaks of magnetite are broader than those of silver, suggesting that the average size of silver nanoparticles is larger than magnetite nanoparticles. It is proved by the approximate values of *d* presented in Table 1. However, calculated values of lattice constant and small size of diffracting domains indicate that obtained magnetite nanoparticles are possibly affected by some microstructural defects. Also, similar results have already been reported and ascribed to the large lattice stress from the interface of Fe_3_O_4_/Ag and the low crystallinity (Huang et al., 2011).

The characteristic PXRD patterns confirmed that the magnetite phase of Fe_3_O_4_ was formed and successively coated with AgNPs, which is consistent with the designed route. However, the presence of SiO_2_ layer was not indicated clearly, probably due to the amorphous nature of the silica shell.

**FIGURE 3:**
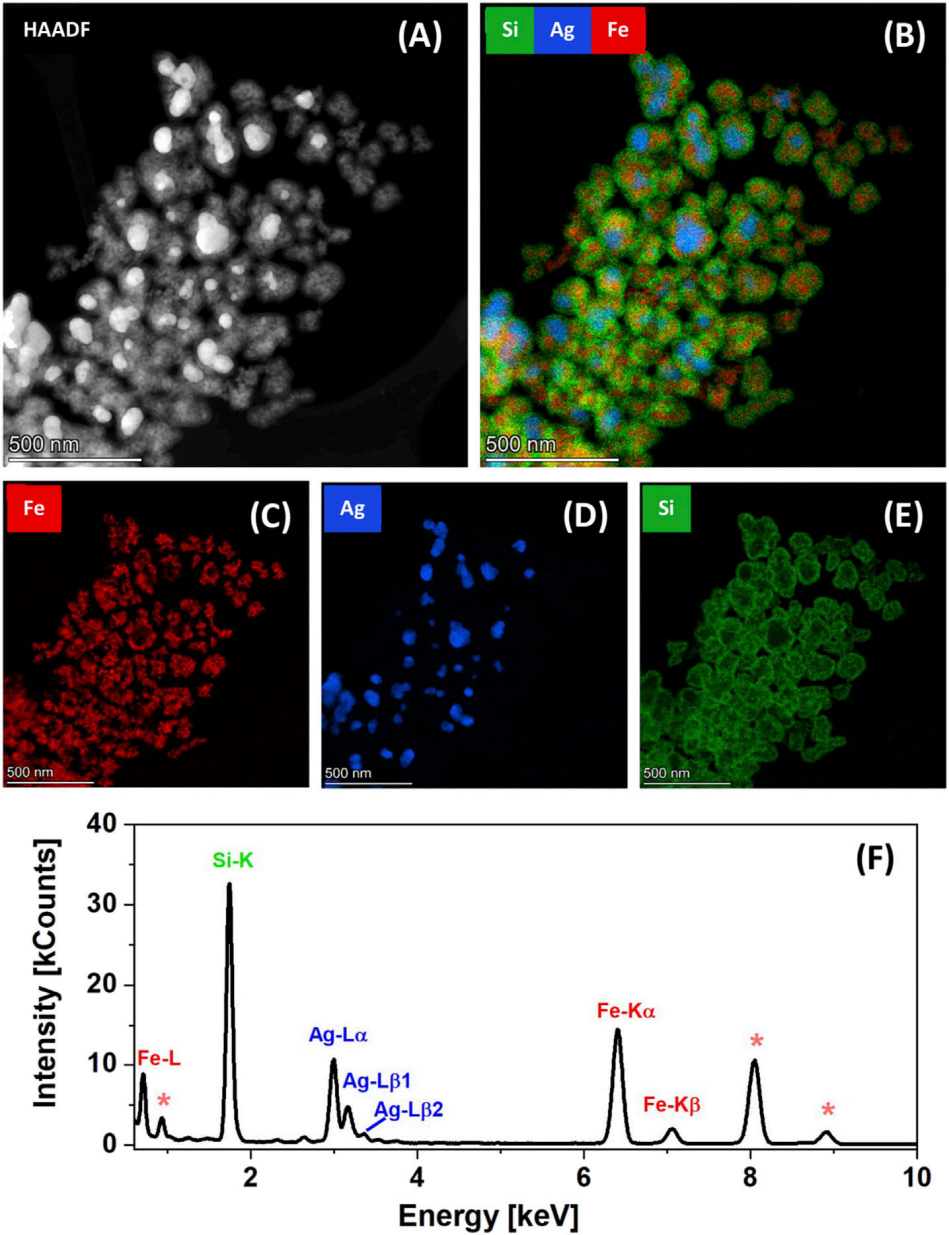
Nanostructural characterization and chemical composition of Fe_3_O_4_/Ag@MES@SiO_2_ nanocomposite: **(A)** STEM-HAADF and corresponding **(B–E)** STEM-EDX elemental mapping images showing the distribution of respectively overlay of Fe+Ag+Si and individual Fe, Ag and Si, with the **(F)** EDX spectrum representative for the area scanned to acquire image presented in part **(A)**; signals from the Cu grid are marked with asterisks.

Further insight into the chemical composition and atomic distribution within the final Fe_3_O_4_/Ag@MES@SiO_2_ nanocomposite was provided by HAADF imaging and elemental composition EDX mapping, performed in STEM mode (see Figure 3). Given that heavy elements appear brighter in HAADF image (signal is proportional to the square of the atomic number), Figure 3A presents the composite of larger, brighter Ag and smaller, darker Fe_3_O_4_ particles. Subsequent EDX mapping of the same region indicates that there are small Fe_3_O_4_/Ag clusters surrounded by a Si-rich layer (see Figure 3B for the overlaid maps, showing the distribution of Fe, Ag and Si). It also demonstrates that the nanoclusters are particularly well-separated (see the spacing between silica coated small assemblies of the nanocomposites in Figure 3B), thus proving a successful reduced self-aggregation of the magnetic component on the here proposed synthetic route.

STEM-EDX images presenting the distribution of iron (Figure 3C), silver (Figure 3D) and silicon (Figure 3E) are also shown. A clear correspondence with HAADF image (Figure 3A) can be noticed, and the widespread presence of SiO_2_ coating, however limited to the particles clusters (Figure 3E). The representative EDX spectrum from the scanned area is shown in Figure 3F, indicating appearance of irons, silver and silicon in the analyzed material. The presence of Cu in the sample was attributed to the use of carbon-coated copper grid for sample deposition (signals of carbon are not seen in the presented range of the EDX spectrum). Preferential adsorption of MES on the AgNPs was also observed, when analyzing spatial distribution of sulfur (see the Supplementary Figure S1 and the discussion in Chapter 2.1).

Our PXRD and STEM-EDX results clearly confirm the formation of SiO_2_-coated small nanoclusters, containing assemblies of Fe_3_O_4_ (magnetite) and Ag nanoparticles, with MES layer adsorbed on the latter. A STEM-EDX visualization of Fe, Ag and Si distribution, compared with STEM-HAADF image for an individual small cluster is presented in Supplementary Figures S2A–D (see also the relevant part of discussion in Chapter 2.2. of Supplementary Material).

**FIGURE 4:**
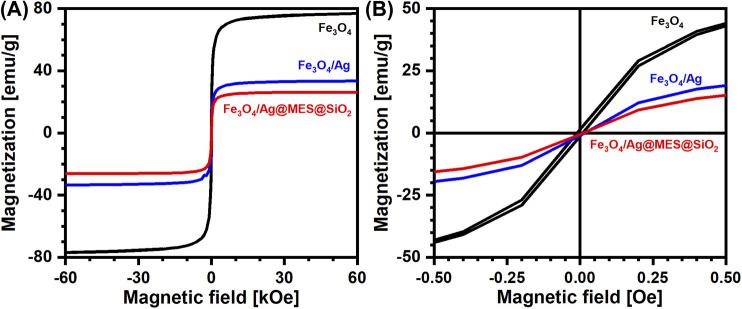
**(A)** Magnetization curves measured by SQUID magnetometry at 300.0 K for the products synthesized at a given preparation step (see legend). **(B)** Enlarged region of the graph shown in **(A)** presenting the zero coercivity under studied conditions.

Magnetic properties of the final nanocomposite and intermediate materials were evaluated with SQUID magnetometry. Figure 4A shows the typical magnetization curves, observed for Fe_3_O_4_ (black line) Fe_3_O_4_/Ag (blue line), and Fe_3_O_4_/Ag@MES@SiO_2_ (red line) particles at 300.0 K. The saturation magnetization (*M*_*s*_) of neat Fe_3_O_4_ nanoparticles, as determined directly from the graph, reached the value of 77.20 ± 0.15 emu/g, while subsequent decoration with AgNPs, adsorption of MES and coating with SiO_2_ layer decreased *M*_*s*_ to 33.56 ± 0.39, 32.64 ± 0.24 and 26.25 ± 0.17 emu/g, respectively. Obviously, the reduced value of *M*_*s*_ after each step of the synthesis is related to the addition of diamagnetic components. Mass percentage concentrations (wt%) of the components at all stages of the reactions were estimated from the changes in the highest observed saturation magnetization. The exact values of all analyzed magnetic properties and wt% values for the intermediate and final products of the synthesis are listed in Table 2 and Supplementary Table S1

**TABLE 2:** Magnetic properties and estimated mass composition of the Fe_3_O_4_/Ag@MES@SiO_2_ and intermediate products based on SQUID.magnetometry measurements.

Sample	Fe_3_O_4_	Fe_3_O_4_/Ag	Fe_3_O_4_/Ag@MES	Fe_3_O_4_/Ag@MES@SiO_2_
Mass of the sample [µg]	1210.2±2.3	383.7±4.4	90.06±0.56	78.14±0.51
Saturation magnetization − 300.0 K, Langevin function fit	72.78±0.85	31.83±0.37	31.24±0.32	25.36±0.23
Mass concentration [%] of the components	$Fe_{3}O_{4}-100$	$Fe_{3}O_{4}-\;43.47\;\pm \;0.51$	$Fe_{3}O_{4}-\;42.28\;\pm \;0.32$	$Fe_{3}O_{4}-\;34.00\;\pm \;0.23$
			Ag− 55.0± 1.2	Ag− 44.22± 0.96
		Ag− 56.53± 0,51	MES− 2.7± 1.2	MES− 2.2± 1.1
				$\mathrm{Si}O_{2}-\;19.6\;\pm 1.5$

It is worth emphasizing that nearly a third of the initial magnetization is preserved the final nanomaterial (see Figure 4A and Table 2), which resulted in its fast motion in the presence of the external magnetic field. This proves a strong magnetic response of the Fe_3_O_4_/Ag@MES@SiO_2_ nanocomposite. On the other hand, the nanoparticles can be easily re-dispersed in suspension after collection with a magnet, already with a slight shaking in the absence of the magnetic field. This feature allows easy manipulation of the nanocomposite, as well as a magnet-assisted controlled assembly of the nanomaterial on the solid substrate prior to the SERS measurements.

Analysis of size distribution of Fe_3_O_4_ NPs (Figure 1C) cannot unequivocally recognize superparamagnetic behavior. At the same time, *zero-field cooling/field heating/field cooling* (ZFC/FH/FC) experiment showed that the utilized Fe_3_O_4_ NPs consist of a substantial amount of superparamagnetic particles (Supplementary Figure S3). We believe that zero coercivity at 300.0 K (see Figure 4B for the enlarged region of interest) is unambiguous evidence for the contribution of a non-negligible population of superparamagnetic particles. These magnetic properties were retained throughout the fabrication of the nanocomposite.

Detailed discussion of the ZFC/FH/FC experiment results and the proof for the partly superparamagnetic nature of the nanomaterial can be found in Chapter 2.3 of Supplementary material.

#### Raman Response of the Nanomaterial

As the proper operation of the system relies on its Raman signal, responses of the nanocomposite before and after covering with SiO_2_ were both verified. Raman results from Fe_3_O_4_/Ag@MES, before silica encapsulation, were collected using the green laser (532 nm). In this case, only the Raman response characteristic to various iron oxide forms was observed (see Supplementary Figure S4). Initially, the Raman bands around 680 and 490 cm^−1^, typical to magnetite (Fe_3_O_4_) (Graves et al., 1988) were noticeable (Supplementary Figure S4A).

However, the broad feature above 330 cm^−1^ may indicate partial transformation to maghemite (γ-Fe_2_O_3_) (Zhang et al., 2013; De Faria et al., 1997). Full oxidation of magnetite to hematite takes place under prolonged illumination with 532 nm laser, as evidenced by the Raman bands at 213, 278, 391, 486, and 689 cm^−1^ (De Faria et al., 1997) (see Supplementary Figure S4B). Similar effect induced by 514.5 nm wavelength was reported for magnetite NPs by Shebanova and Lazor (Shebanova and Lazor, 2003). Surprisingly, SERS signature of MES molecules was untraceable. We believe that here presented Fe_3_O_4_/Ag nanocomposite exhibits non-negligible absorption at 532 nm, leading to the phase transition from magnetite to hematite and very likely a related local heating, resulting in thermal degradation of the thiolate layer. Comparable activity as nano-heaters was demonstrated for hybrid gold-iron oxide nanoparticles, only in the region exposed to 532 nm laser radiation (Hoskins et al., 2012).

**FIGURE 5:**
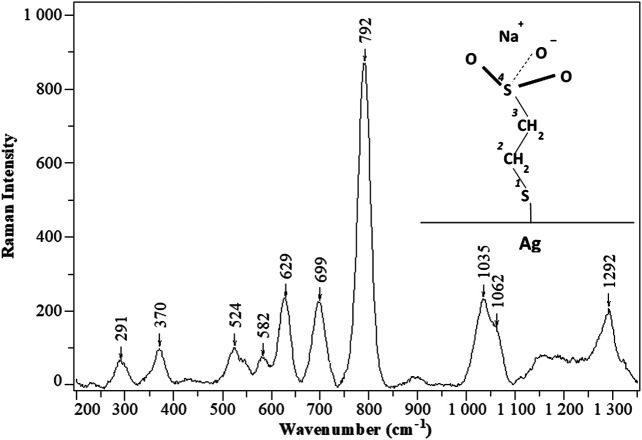
Typical SERS spectrum (λ_exc_=632.8 nm, 3 × 10 s acquisition time, laser power on the sample around 0.75 mW) of MES adsorbed on Fe_3_O_4_/Ag nanocomposite. The spectrum was baselined for the clarity of presentation. Inset: schematic structure of the MESNa molecule adsorbed on silver in *trans* conformation.

Change of the excitation wavelength to 632.8 nm resulted in the appearance of SERS spectrum characteristic of MES molecules (Figure 5). The assignment of the most prominent SERS bands to MES molecular vibrations is given in Table 3, while the atom numbering is shown in the inset. A relative amount of the rotational conformers in the MES layer can be deduced from the SERS intensity ratio of the bands corresponding to ν(^2^C−^1^S) vibrations characteristic of *trans* (near 700 cm^−1^) and *gauche* (around 630 cm^−1^) conformers. The two components of the doublet, visible around 1036 and 1065 cm^−1^ in Figure 5, were attributed to the symmetric stretching vibrations of MES molecules with SO_3_^–^ groups respectively freely solvated or forming contact ion pairs with metal cations (here: Na^+^) (Kudelski et al., 2002). The changes in the intensity of this doublet were successfully used for the SERS-based quantitative detection of various metal cations (Piotrowski and Bukowska, 2015). Hence, analysis of SERS spectrum of MES layer provides insight into the structural features of the thiolate under studied conditions. However, here we were mostly interested in the use of the most intense SERS bands of MES around 790 cm^−1^ (whose intensity is much less sensitive to the environmental conditions) to probe the efficiency of the SERS enhancement.

**TABLE 3:** Vibrational assignment of the most intense SERS bands of MES on Fe_3_O_4_/Ag nanocomposite.

wavenumber/cm^−1^	Assignment
291	ν(Αg–^1^S)
629	ν(^2^C–^1^S)_*gauche*_
699	ν(^2^C–^1^S)_*trans*_
792	ν(^3^C–^4^S)
1035 and 1062	ν_s_(SO_3_^−^)
1292	ν_as_(SO_3_^−^)

#### Decoration of Magnetite With AgNPs

Successful synthesis of well-separated Fe_3_O_4_/Ag@MES@SiO_2_ nanocomposite consisted of several steps (Figure 1A) and the starting point was the fabrication of Fe_3_O_4_ NPs through co-precipitation method (*Synthesis of Fe_3_O_4_ Nanoparticles* in *Materials and Methods*). *Each subsequent process had to be optimized to ensure that the best conditions were applied. Decoration of magnetite nanoparticles with silver nanostructures was performed by* in situ *reduction of silver ions (originating from the AgNO_3_ solution introduced into the reaction mixture) with* *n*-ButNH_2_. Our initial conditions were based on the protocol applied for much larger spherical Fe_3_O_4_ NPs (near 300 nm in diameter), described in the literature (Kim et al., 2010). For effective coating with AgNPs, we decided to increase both the amount of magnetite powder – from the original 1 mg/10 ml to 2 mg/5 ml – and concentration of silver ions – from 8 to 16 mM. Other physical conditions of the reaction were not changed compared to the literature procedure (Kim et al., 2010). In the initially synthesized material, there was insufficient amount of silver nanostructures attached to magnetite NPs in the such prepared Fe_3_O_4_/Ag nanocomposite, while existing sparse AgNPs were strongly non-uniform in size, with a strong contribution of very large and randomly distributed nanoobjects (TEM images not shown) To address these issues, we introduced sonication of the bare Fe_3_O_4_ NPs suspension prior to the Ag^+^ ions reduction step, which significantly increased the amount of the silver clearly attached to magnetite, as confirmed with TEM analysis. Sonication stage was therefore further applied in all attempts described below, aiming at optimization of the structural features of the fabricated Fe_3_O_4_/Ag nanocomposite towards its performance in SERS spectroscopy.

Our preliminary results clearly showed a few obstacles to overcome, namely high total content of the AgNPs in the sample, excessive amount of the large silver nanostructures, and their high size polydispersity. In order to find the best conditions, varying amounts of Fe_3_O_4_ NPs in a powder form (1 or 2 mg per 5 ml of water) and concentration of silver nitrate (2, 4, 8 or 16 mM) were examined in the step of decoration with AgNPs. Interplay between these two experimental factors was expected to affect the size, distribution and amount of formed silver nanostructures. Additionally, for selected variants of synthesis, the effect of the change in molar ratio of Ag^+^ to *n*-ButNH_2_ from 1:1 to 5:1 was investigated, as it was expected to increase the efficiency of the silver nucleation process.

TEM images of the intermediate nanocomposites obtained during optimization procedure (Supplementary Figures S5, S7), together with their respective histograms displaying the size distributions of AgNPs (see respectively Supplementary Figures S6, S8) and a detailed discussion of the results can be found in Chapter 2.5 of Supplementary Material. Supplementary Table S2 presented in the same chapter summarizes the values of average size with the respective relative standard deviation (RSD) as a function of the being optimized parameters of synthesis.

The nanocomposite fabricated with 8 mM AgNO_3_ (using 2 mg/5 ml of magnetite NPs and 1:1 Ag^+^ to *n*-ButNH_2_ molar ratio) appeared the most appealing for the prospective application in SERS spectroscopy, due to its promising mean size 72.5 ± 32.7 nm and good separation of the silver nanostructures (see Supplementary Figures S7B,B’), preventing appearance of random hot spots.

Interestingly, when the mean value of the silver particle size increases, the absolute value of SD also increases (see Supplementary Table S2), but while for the data set presented in Supplementary Figures S5, S6 the value of RSD is relatively constant (30–33%), for the results shown in Supplementary Figures S7, S8, starting from 4 mM AgNO_3_ concentration – the higher the mean size, the higher the RSD is observed (33% for 2 and 4 mM, 45% for 8 mM and 49% for 16 mM). Apart from one sample analyzed in Supplementary Figure S5 (shown in part D), the other ones were prepared at constant concentration of silver(I) nitrate, while in Supplementary Figure S7 this was the only varying parameter. This result indicates that concentration of AgNO_3_ influences not only the mean size of the formed silver nanoparticles but also their size distribution.

To perform a further, qualitative evaluation of SERS performance of the fabricated materials, SERS response for the Fe_3_O_4_/Ag nanocomposites obtained with selected protocols were collected and analyzed. Namely, only the materials for the previously established amount of magnetite NPs and ratio of silver ions and reducing agent equal respectively to 2 mg/5 ml and 1:1 were used, while the impact of initial concentration of silver ions was investigated. The values of the normalized (see *Normalization Procedure of SERS Intensity* of Material and Methods for the details) mean SERS intensity and (R)SD are listed in Supplementary Table S3. This preliminary evaluation was carried out for the MES molecules adsorbed for 2–5 h, while effect of time of adsorption of Raman tag on SERS response is described in more details in *Effect of the MES Adsorption Time on SERS Response*. Note that for all the used amounts of the silver ions we examined at least two various areas of the sample, distant from each other.

The observed trend of increasing intensity of the SERS signal with the varying concentration of AgNO_3_ is as follows: 2 mM < 4 mM < 16 mM < 8 mM. The SERS response of 8 mM AgNO_3_ is nearly one order of magnitude stronger, than those of 2 and 4 mM. Moreover, the magnitude of SD for the lowest used amount of silver ions (2 mM) is in the range of the measured signal. The latter results from the strongly non-uniform spatial distribution of the surface enhancement across the sample for the Fe_3_O_4_/Ag nanocomposite synthesized according to this protocol. This conclusion is consistent with the observation of relatively small population of AgNPs in TEM images for the sample fabricated with the use of 2 mM silver nitrate (see Supplementary Figures S7D,D’)*.*

The value of relative standard deviation (RSD) is substantially improved when the concentration of AgNO_3_ is higher than 2 mM. The typical value of RSD was comparable (around 30–35%) for the 8 mM and 16 mM AgNO_3_, however for the former sample even the area with the RSD below 25% was found, while for the latter RSD as high as 44% (see Supplementary Table S3). Both discussed materials revealed very good reproducibility of the mean value of SERS intensity, independent on the selected region of the sample (see Supplementary Table S3). However, the use of 8 mM AgNO_3_ offered higher SERS intensity, advantageous regarding the subsequent silica coating, expected to reduce the original SERS signal.

These results are in good agreement with our previous conclusions, derived from the analysis of the size and morphology of the samples, based on TEM imaging.

#### Effect of Polyelectrolytes on Nanocomposite Aggregation and Coating With SiO_2_ Layer

**FIGURE 6:**
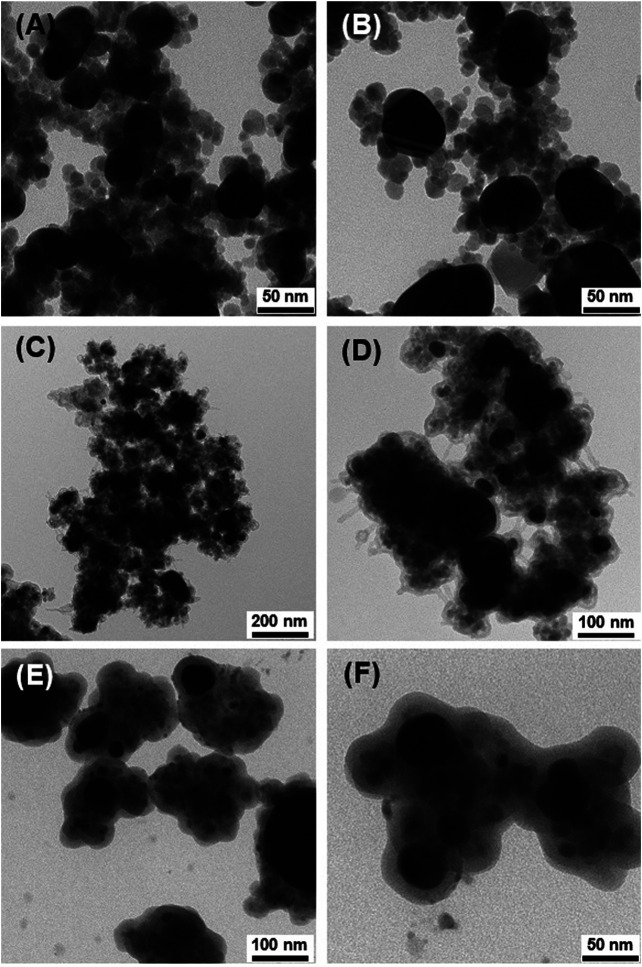
TEM images of Fe_3_O_4_/Ag nanocomposite synthesized with 2 mg/5 ml of magnetite NPs, 1:1 Ag^+^ to *n*-ButNH_2_ molar ratio and 8 mM AgNO_3_ **(A)** and next subjected to Stöber reaction: **(B)** without MES and without PE, **(C,D)** with MES and with PE; **(E,F)** with MES and without PE layer.

The final step of the synthesis was coating the Fe_3_O_4_/Ag@MES composite with a silica layer. TEM image of the nanomaterial demonstrated in *Decoration of Magnetite With AgNPs* as exhibiting the best SERS performance (synthesized with 2 mg/5 ml of magnetite NPs, 1:1 ratio of [Ag^+^]/[*n*-ButNH_2_] and 8 mM AgNO_3_) before the modified Stöber reaction is shown in Figure 6A. The formation of a SiO_2_ layer did not proceed when MES layer was not absorbed on the nanocomposite (see Figure 6B). Here used conditions of Stöber method were rather mild, therefore, in order to accomplish successful silica coating at room temperature and protect our SERS tag, we decided to deposit additional layer of the polyelectrolytes (PE) following MES adsorption, prior to SiO_2_ growth (Kim et al., 2008). In this case, a continuous layer of SiO_2_ was formed, but we noticed significant spontaneous aggregation of the nanomaterial during synthesis of Fe_3_O_4_/Ag@MES@SiO_2_ according to this protocol (Figures 6C,D). Very large aggregates of the nanocomposite, encapsulated in a silica shell are clearly visible for these experimental conditions. We discovered that much smaller clusters are formed when the silica layer was grown directly after MES adsorption, without coating with PE layer (Figures 6E,F). Apparently MES molecules facilitated re-dispersion of Fe_3_O_4_/Ag and simultaneously introducing additional priming molecules was not necessary to prepare well separated, silica-coated small nanoclusters, promising for their SERS activity.

### SERS Performance

#### Effect of the MES Adsorption Time on SERS Response

**FIGURE 7:**
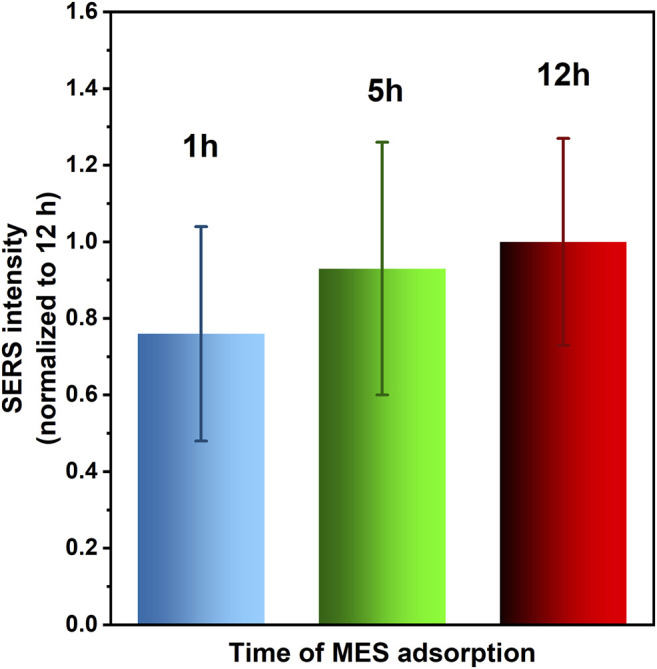
Average intensity of MES SERS band around 790 cm^−1^ vs time of MES adsorption (with error bars showing the RSD values). The results are normalized to the SERS intensity measured for 12 h time. Typically 25–36 spots localized across 60–80 μm x 100–120 μm rectangular area were analysed in each case.

We investigated the effect of the MES adsorption time on the SERS response of the system. For that purpose 2 mg Fe_3_O_4_/Ag nanocomposite was immersed in 1 ml of 1 mM MES solution for 1 h, 5 h or 12 h. The most intense SERS spectra were observed after 12 h of MES adsorption, while the average SERS intensity for 5 h and 1 h reached respectively 0.93 and 0.76 of the value determined for the longest time of immersion. The values of RSD and thus SERS signal reproducibility was less affected by the time of Raman tag adsorption, being in a range of 27–33%. To show these in more details, a plot of averaged intensities of SERS peak around 790 cm^−1^ normalized vs the SERS intensity of the same band observed for 12 h time is presented in Figure 7. The RSD values of SERS intensities for 12 h, 5 h, and 1 h time of adsorption are 27%, 33% and 28%, respectively.

**FIGURE 8:**
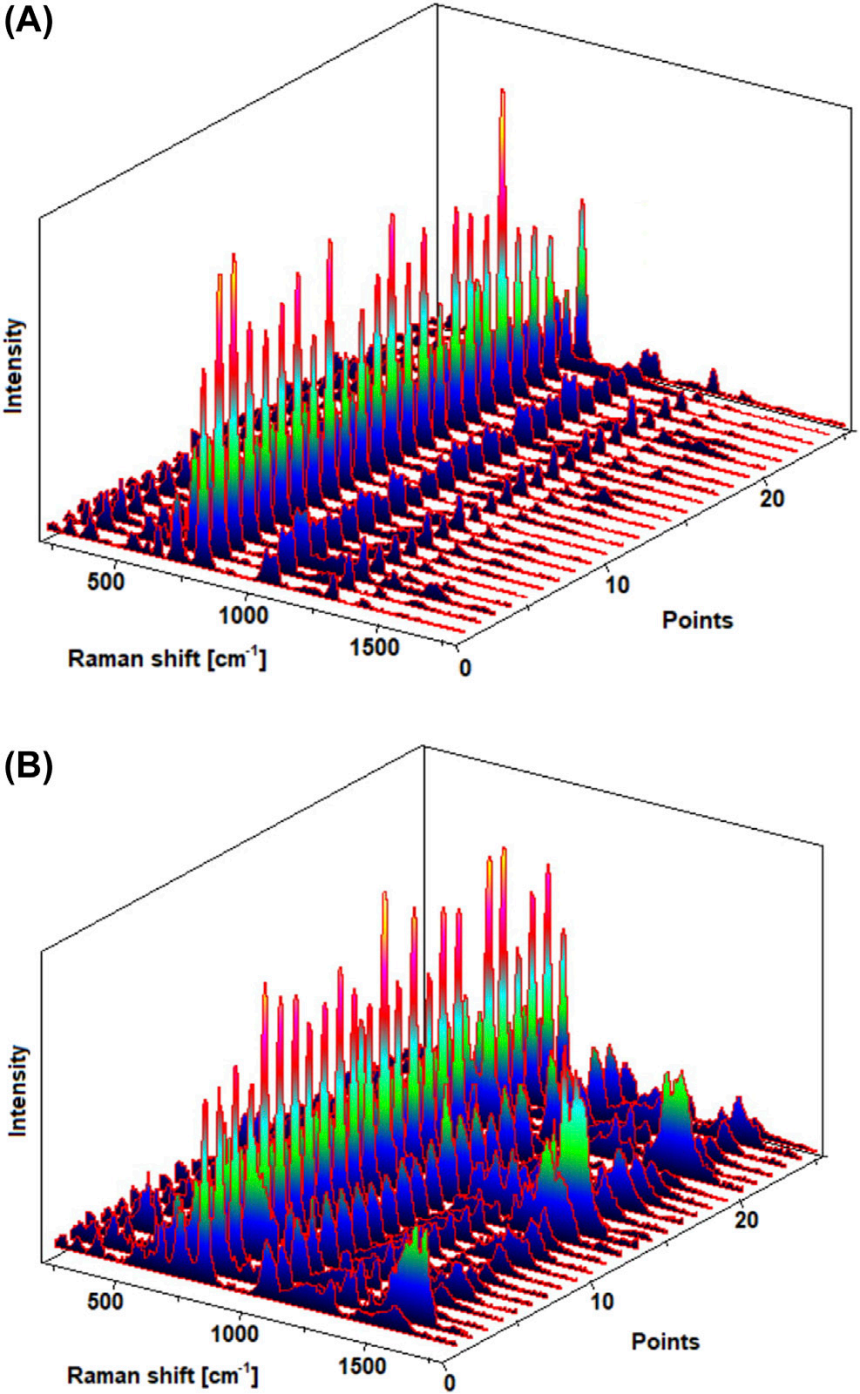
3D display of series of SERS spectra (excitation line 632.8 nm, 3 × 10 s acquisition time for each spectrum, laser power on the sample around 0.75 mW) of MES on Ag/Fe_3_O_4_ adsorbed for 12 h **(A)** and 1 h **(B)**, collected from various locations of the sample (listed as points). For part **(B)** the characteristic “carbon fluctuations” are clearly visible in the spectral range above 1300 cm^−1^.

More systematic analysis of the Raman maps showed that MES monolayer grown for only 1 h undergoes thermal decomposition more easily than this formed for 12 h. This was deduced from the fluctuations of SERS signal captured in the acquired spectra, arising from individual carbon species (Kudelski, 2006), generated upon exposure to the laser beam, additionally enhanced by plasmonic effect. SERS signal was much more stable when longer time of MES adsorption was applied, as demonstrated by the contribution of temporal fluctuations of some SERS peak intensities (not attributable to MES vibrational modes), as well as varying peak/peak intensity ratios (see Figure 8 for comparison of the 1 h and 12 h adsorption time). Although the MES monolayers on silver were reported to be very stable (even after storing in the electrolyte solution), apparently this adsorption behavior is feasible for more self-organized and defect-free monolayers, viable for long adsorption time [24 h and 10 mM solution of MESNa was applied by Kudelski (Kudelski, 2002)].

Taking into account all these observations, we decided to proceed further with the MES layer grown for 12 h, as this one provided the highest SERS activity, reasonable spatial reproducibility of the SERS signal and improved thermal stability among studied samples.

#### Influence of Silica Coating and Method of Purification

Silica-encapsulation of Fe_3_O_4_/Ag@MES is expected to substantially reduce the SERS signal of the MES tag molecule. In principle, the larger the thickness of the silica layer, the larger the loss of the SERS intensity can be predicted. Thickness of the shell affects the magnitude of electromagnetic enhancement, as the latter decays rapidly with distance (*r*) from the SERS-active NP, scaling with the *r* ^*-10*^ for spherical particles. Therefore, the largest values of the electromagnetic enhancement factor are found in the range of a few nanometers from the substrate surface (Stiles et al., 2008). Overly thick coating can result in blocking the incident and scattered photons, eventually even suppressing the SERS signal (Li et al., 2017). On the other hand, shell of small thickness can exhibit discontinuities and its isolating function will be compromised (Li et al., 2013).

The effect of the purification procedure on the SERS properties of the Fe_3_O_4_/Ag@MES@SiO_2_ nanocomposite was additionally examined after the last stage of the synthesis. We tested magnetic field-assisted washing, using two different solvents: rinsing the final product consecutively with water, ethanol, and water (WEW) or three times with water only (3xW).

STEM images shown in Supplementary Figure S9 (Chapter 2.6 of Supplementary Material) demonstrate that there is no obvious effect of the purification method on the size of silica shell, but the WEW order resulted in formation of the strongly agglomerated nanocomposite particles, attached together via the SiO_2_ layer (see Supplementary Figures 9A,B). On the other hand, use of only water for purification (3xW) led to emergence of nicely separated small SiO_2_-coated clusters (see Supplementary Figures S9C,D). Bare Fe_3_O_4_/Ag particles can hardly be found, while silica-embedded nanoparticles composed purely of Fe_3_O_4_ can be easily noticed for both WEW and 3xW washing procedure.

In the next step, SERS activity was evaluated as a function of the method of post-synthesis purification (Raman maps collected from the samples can be found in Supplementary Figure S10 alongside the discussion of the Raman mapping results in Chapter 2.6 of Supporting Material).

The change of the SERS signal of the Fe_3_O_4_/Ag@MES upon SiO_2_ encapsulation was evaluated for both examined methods of purification, using the integral intensity of the SERS band of MES around 790 cm^−1^. The parameters describing SERS properties of the examined nanomaterials are presented in Supplementary Figure S11. For the 3xW washing procedure around 26% of the original SERS intensity was preserved after silica coating, comparing to only 15% in case of WEW procedure. Moreover, the RSD value was somewhat improved upon silica coating, changing from 30% for bare Fe_3_O_4_/Ag/MES to 19% for Fe_3_O_4_/Ag/MES/SiO_2_ nanocomposite, solely for the 3xW method of purification.

In conclusion, all the results presented in this chapter clearly prove the superiority of the 3xW washing protocol, considering the separation of the nanoclusters, as well as thermal stability of the final Fe_3_O_4_/Ag@MES@SiO_2_ nanocomposite and its SERS response.

#### Impact of the Magnetic Field-Assisted Assembly

**FIGURE 9:**
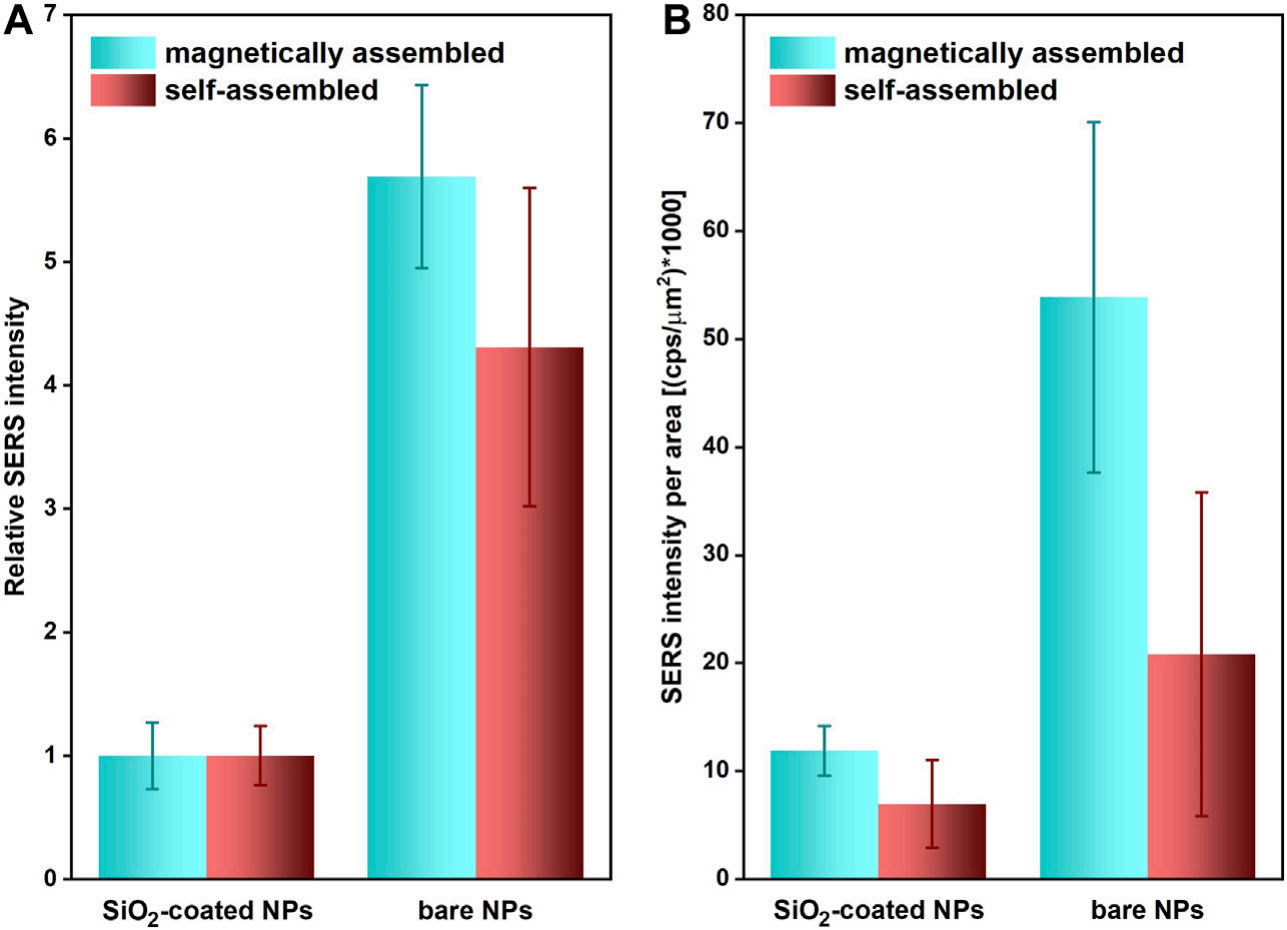
**(A)** Changes of the average intensity of MES SERS band around 790 cm^−1^ (with error bars showing the RSD values) for magnet- and self-assembled (ordered region) samples of bare and SiO_2_-coated Fe_3_O_4_/Ag@MES NPs onto glass substrate. Relative intensity corresponds to SERS signal with respect to this observed for the magnetically assembled silica-coated NPs (normalized to unity). The values of RSD were also recalculated according to this procedure. **(B)** Average intensity of MES SERS band around 790 cm^−1^ (with error bars showing the RSD values) divided by the examined area for magnet- and self-assembled (disordered region) samples of bare and SiO_2_-coated Fe_3_O_4_/Ag@MES NPs onto glass substrate. Typically 25 spots localized across area not smaller than 6300 μm^2^ area were analysed in each case.

Use of external magnetic field in case of plasmonic-magnetic nanomaterial not only offers efficient separation of nanocomposite and/or analyte from the complex mixture but can also be beneficial for SERS detection, as two-dimensional films can be obtained in a more reproducible and scalable manner. Therefore, we evaluated the impact of the external magnetic field on the SERS performance of Fe_3_O_4_/Ag@MES and Fe_3_O_4_/Ag@MES@SiO_2_ by monitoring the intensity of the MES band around 790 cm^−1^ for the nanocomposites deposited onto a glass substrate (see Figure 9A). Surprisingly, when choosing a nanomaterial-rich region of the sample, a magnetic field-assisted assembly leads to the decreased average SERS intensity (by roughly 25%) and larger values of RSD (nearly two times) for the bare Fe_3_O_4_/Ag@MES. Nevertheless, this effect was observed only for the high values of laser power. Results under the high-power conditions, presented with respect to those acquired for the magnetically assembled Fe_3_O_4_/Ag@MES nanocomposite (normalized to unity), are presented in Figure 9A.

At the same time, SERS response was nearly identical—irrespective of the presence of the external magnetic field at the sample preparation step—when the power of the laser beam was reduced 10 times (data not shown). This trend suggests that most likely electromagnetic hot spots exhibiting too high plasmonic activity are formed under high-power illumination, which results in the reduced and more random SERS signal across the surface.

Investigation of the optical images of representative areas for the two discussed samples (see Supplementary Figure S12 in Chapter 2.7 of Supplementary Material) shows substantial differences at the level of order and roughness of the formed films. Strongly non-uniform and rough layer was obtained without the external magnetic field, which can be attributed to the random aggregation of the nanocomposite during solvent evaporation (Supplementary Figure S12A). On the other hand, a smooth and homogenous film was found for magnetic-assembled bare nanocomposite (Supplementary Figure S12B). These observations encouraged us to choose another 25 spots across the non-uniform and strongly disordered area of the substrate, which was a typical pattern for the sample deposited in the absence of the external magnetic field. The scanned areas for the all studied nanocomposites (prepared with or without the magnet) ranged from around 6200 to 8100 μm^2^, while the exact values for the examined samples can be found in the legend to Supplementary Figure S12. Next, using these values we compared the SERS signal intensity per scanned unit area for the magnetic-assembled and self-assembled samples (Figure 9B), which showed the significant advantage of the specimen when magnetic field was applied, both in terms of the average intensity and RSD values. The latter deteriorated from 22 to 42% for Fe_3_O_4_/Ag@MES@SiO_2_ and from 30 to 72% for Fe_3_O_4_/Ag@MES when magnetic field was eliminated in the assembly step. As can be seen in Figure 9B, the values of the average SERS intensity [in (cps)] divided by examined area [in (μm^2^)] and multiplied by 1000 (for the clarity of presentation) are improved by a factor of 1.72 and 2.58 for respectively silica-coated and bare nanocomposite, when external magnet was used for assembly.

The most likely explanation of this behavior is that the external magnetic field guided assembly of the nanocomposite allows the preparation of plasmonic hot spots of high density. Similar properties have been already reported in the literature for the gold-coated magnetic nanoparticles and ascribed to their magnetic field controlled accumulation (Shibusawa et al., 2019).

Silica encapsulation of the Fe_3_O_4_/Ag@MES mostly eliminates the effect of the external magnetic field on the SERS response of the nanocomposite (Figure 9A). Here obtained silica shells are not ultrathin, thus we attribute this behavior to the assembly limited by the thickness of the SiO_2_ layer. Consequently, secondary hot spots at the junctions of the nanoparticles are very likely not generated and therefore there is no impact of the magnetic field on the observed SERS intensity and RSD values.

### Potential Applications

#### Protective Role of Silica Shell

**FIGURE 10:**
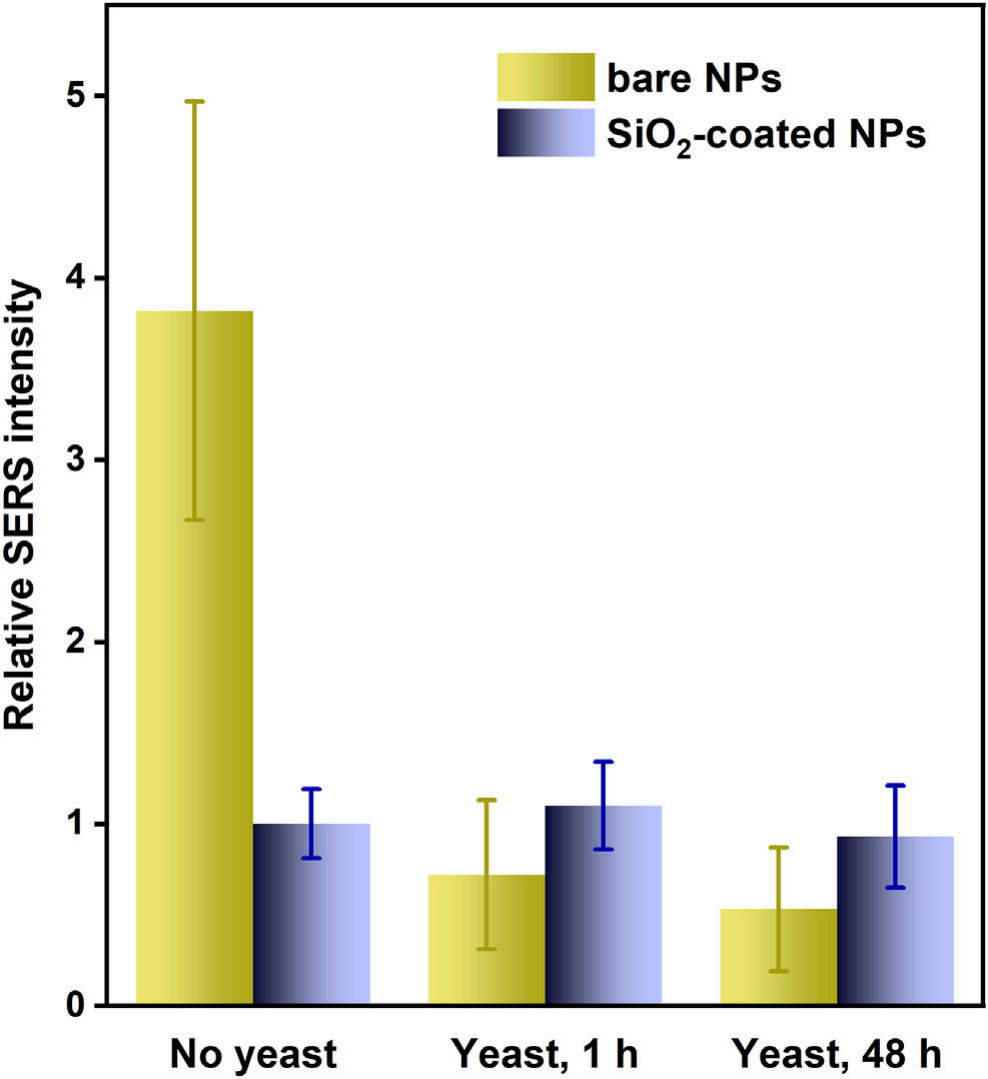
Changes of the average intensity of MES SERS band around 790 cm^−1^ (with error bars showing the RSD values) for bare and SiO_2_-coated Fe_3_O_4_/Ag@MES NPs upon exposure to the suspension of yeast cells for 1 h and 48 h. Typically 20–25 spots localized across 140 μm × 120 μm rectangular area were analysed in each case. Relative intensity corresponds to SERS signal with respect to this observed for the silica coated NPs before contact with yeast (normalized to unity). The values of RSD were also recalculated according to this procedure.

The SiO_2_ shell applied in this work is not only beneficial for the reduced self-aggregation of the magnetic component and the Fe_3_O_4_/Ag@MES nanocomposite but also prevents the triggered dissolution of the encapsulated nanoparticles, thus improving their chemical stability. We demonstrated this protective role of silica coating by mixing colloidal nanocomposite with the yeast suspension and comparing the temporal evolution of SERS signal for SiO_2_-coated and bare Fe_3_O_4_/Ag@MES nanomaterials. The results are presented in Figure 10, where SERS intensity observed for Fe_3_O_4_/Ag@MES@SiO_2_ not exposed to yeast was taken as a reference (and normalized to unity). It is clearly seen that the intensity of the original SERS signal is reduced by more than 75% (compare the height of the representative yellow bars in Figure 10 for bare nanocomposite, already after one hour of contact with the yeast suspension). Moreover, SERS response of the uncoated nanocomposite after treatment with yeast cells becomes even weaker than this characteristic for undisturbed by yeast Fe_3_O_4_/Ag@MES@SiO_2_ nanomaterial (compare the height of the respective yellow and navy blue bars in Figure 10). On the other hand, SERS spectra are barely affected both after 1 and 48 h of interaction with yeast, when the silica shell is present within the nanocomposite. Only the RSD is slightly deteriorated, changing from around 20% (initially and after 1 h) to 30% (after 48 h). Interestingly, prolonged exposure of bare Fe_3_O_4_/Ag@MES to yeast cells does not lead to a significant further decay of its characteristic SERS intensity, as evidenced by only slightly decreased SERS signal after 48 h, compared to this observed after 1 h (see Figure 10).

We believe that the drop of the SERS intensity in the case of uncoated Fe_3_O_4_/Ag@MES arises from the decomposition of silver component of the nanocomposite upon contact with yeast cells and resulting gradual loss of their plasmonic activity. This was further confirmed by the collected extinction spectra, which showed that surface plasmon resonance peak—observed initially around 400 nm—disappears quickly already after 1 h of mixing with yeast suspension for the bare nanoparticles (Supplementary Figure S13 in Chapter 2.8 of Supplementary Material). After 48 h there is still some residual extinction (dark violet curve in Supplementary Figure S13), explaining a noticeable weak SERS signal for this time of contact with yeast suspension (Figure 10). These results prove an important protective function of SiO_2_ coating layer, which should be feasible also in more corrosive environment [except the strongly alkaline media, where some other transparent oxide coatings can be used instead of silica (Abdulrahman et al., 2016)]. So far, a similar shielding role of silica shell was demonstrated only for purely plasmonic nanoparticles (Koła̧taj et al., 2017), while according to our knowledge this is the first time when the plasmonic-magnetic nanocomposite is shown to be successfully protected in the presence of the suspension of yeast cells. The magnetic component was also secured by SiO_2_ shell, as the nanomaterial was still easily collected with the external magnetic field.

#### Authenticating or Labeling Objects by Means of SERS Nanotags

**FIGURE 11:**
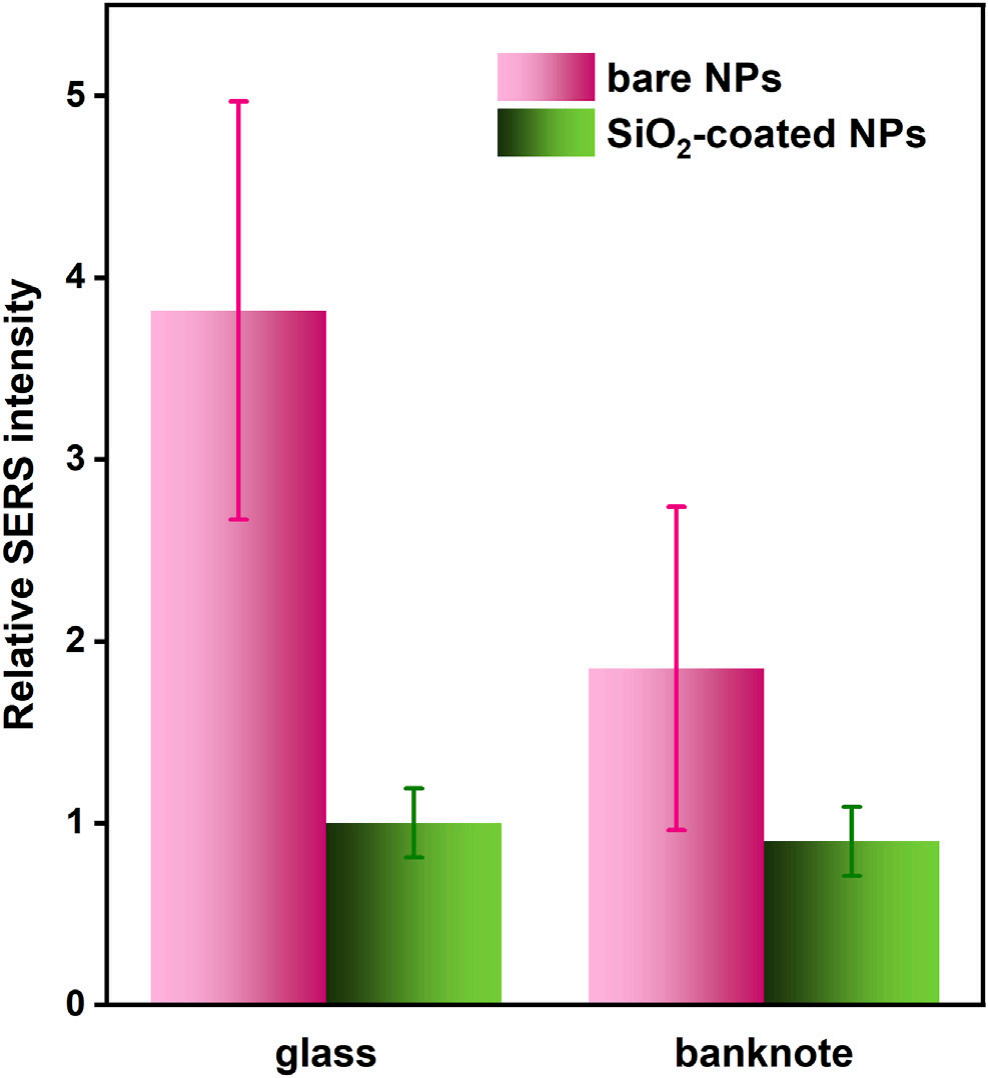
Comparison of the average intensity of MES SERS band around 790 cm^−1^ (with error bars showing the RSD values) for bare and SiO_2_-coated Fe_3_O_4_/Ag@MES NPs deposited. Typically 20–25 spots localized across 140 μm × 120 μm rectangular area were analysed in each case. Relative intensity corresponds to SERS signal with respect to this observed for the silica coated NPs on glass (normalized to unity). The values of RSD were also recalculated according to this procedure.

SERS nanotags are excellent anti-counterfeiting and/or marking labels, due to their very high sensitivity of detection, unique molecular signature, and ability to quench the fluorescence (Lai et al., 2015; Li et al., 2016). These features provide respectively fast optical response, high-encoding capacity due to multiple SERS spectral features, and functioning in a complex matrix of real-life items.Both silica-coated and bare Fe_3_O_4_/Ag@MES were deposited on a 20 PLN (Polish zloty) banknote and their SERS responses were collected and compared, in order to verify their applicability for the authentication/marking purposes and effect of the silica shell on the latter (see Figure 11).

SERS signal is nearly identical in terms of average intensity and its RSD values when comparing deposition on glass and banknote for SiO_2_-protected nanotags. In contrast, both of these SERS signal parameters were deteriorated, when bare Fe_3_O_4_/Ag@MES nanoparticles had been placed on the banknote. Average SERS intensity decreased nearly two times, while the RSD increased from 30% to almost 50%; at the same time, the latter was undisturbed for silica-coated nanocomposite, amounting to approximately 20%. Average SERS intensity on the glass substrate was around four times higher for the uncoated Fe_3_O_4_/Ag@MES nanomaterial than for the SiO_2_-modified one, while on the banknote the intensity difference was only twofold. We believe that these deteriorated parameters of SERS signal observed exclusively for the bare NPs on the banknote comparing to glass are due to the additional interactions of the light with a complex matrix of the banknote material, which apparently must be somehow suppressed by the presence of SiO_2_ layer. Recalling improved chemical stability of the silica encapsulated nanocomposite demonstrated in *Protective role of silica shell*, their stable SERS response and here shown narrowed gap between their SERS performance and that of uncoated ones, all those features make them attractive as nanotags for a variety of real-life surfaces, including labeling and authentication of different objects.

## Conclusion

To sum up, we present a facile and efficient way of preventing excessive self-aggregation in magnetic-based nanomaterials by applying a monolayer of 2-mercaptoethanesulfonate (MES) within Fe_3_O_4_/Ag@MES@SiO_2_ nanocomposite. MES promotes the growth of the silica layer in a controllable manner, resulting in the fabrication of well-separated SERS-active magnetic-plasmonic clusters, simultaneously providing a reliable SERS signal of MES as a Raman tag.

Obtained nanocomposite exhibits chemical resistance in aggressive conditions, owing to encapsulation of the Raman tag within the SiO_2_ shell. That secures a reliable and stable SERS response, that is highly desired e.g. in bioapplications. This aspect is additionally supported by the non-toxicity of MES molecules.

We also observed a significantly higher density of hot spots upon magnetic assembly of the nanomaterial, more pronounced for the bare Fe_3_O_4_/Ag@MES. More homogenous distribution of the nanoparticles can be achieved for the silica-covered nanocomposite, for which absolute SERS intensity becomes irresponsive to the external magnetic field.

It is worth pointing out that the proposed procedure can be extended to other magnetic-based nanomaterials, where it is essential to reduce the inter-cluster attraction. Provided that the MES molecule can be adsorbed on the surface, we can apply here proposed synthesis conditions to improve the quality of various nanosystems for many applications. Magnetic and magnetic-derived nanoparticles are already found useful in a wide range of research areas, including information coding, safety nanosystems, sensing, and biodetection, and this scope can be extended by boosting the parameters of the nanocomposite.

What is more, the proposed approach can be customized and applied to various studies for at least two more reasons. One is a possibility to further extend the system by functionalization of the outer silica shell. This allows for assembling elaborate architectures, tailored for particular purposes e.g., aiming at certain cell lines. Another aspect is the feasibility of introducing different Raman and/or fluorescence tags alongside MES to guarantee multi-modal detection.

Finally, our system is demonstrated to be applicable as SERS-active nanotags in real-life samples (i.e., a banknote) which concomitantly may be relevant in surface analysis. All in all, we presume that our findings will stimulate the development of magnetic systems by enhancing their quality and eventually broadening their usability.

## Data Availability

The raw data supporting the conclusions of this article will be made available by the authors, without undue reservation.
