# Supplementary material for: Reduced Self-Aggregation and Improved Stability of Silica-Coated Fe3O4/Ag SERS-Active Nanotags Functionalized With 2-Mercaptoethanesulfonate
Source: Front Chem. 2021 Jun 16;9:697595. doi: 10.3389/fchem.2021.697595 (PMC8241903; doi:10.3389/fchem.2021.697595)
Supplement: Supplementary file 1 [file DataSheet1.pdf]

SUPPLEMENTARY MATERIAL

Reduced self-aggregation and improved stability of silica-coated Fe_3O_4/Ag SERS-active nanotags functionalized with 2-mercaptoethanesulfonate

Maria Żygieło[1], Piotr Piotrowski[1], Marcin Witkowski[1], Grzegorz Cichowicz[1], Jacek Szczytko[2] and Agata Królikowska[1]

[1] *Faculty of Chemistry, University of Warsaw, Pasteura 1, 02-093 Warsaw, Poland*
[2] *Institute of Experimental Physics, Faculty of Physics, University of Warsaw, Pasteura 5, 02-093 Warsaw, Poland*

TABLE OF CONTENTS

1. Materials and methods **2**

1.1 SQUID magnetometry - measurement uncertainties **2**

1.2.Temperature-dependent measurements of magnetization **3**

2. Results and discussion **4**

2.1 Preferential adsorption of MES on the AgNPs within Fe_3O_4/Ag@MES@SiO_2 visualized with STEM-EDX **4**

2.2 STEM-HAADF and STEM-EDX images of an individual cluster of Fe_3O_4/Ag@MES@SiO_2 **5**

2.3 Zero-field cooling/field heating/field cooling (ZFC/FH/FC) experiment and evidence for population of superparamagnetic Fe_3O_4 NPs **6**

2.4 Raman characterization of Fe_3O_4/Ag@MES using 532 nm laser **10**

2.5 Characterization of the intermediate products during the optimization of the synthesis of nanocomposites **11**

2.6 Impact of method of purification on the structural and SERS properties of the final product **19**

2.7 Effect of magnetic field on assembly of bare and silica-coated nanocomposite **23**

2.8 Temporal evolution of UV-Vis spectra upon mixing with yeast for bare nanocomposite **24**

3. References **24**

1. Materials and methods

1.1 SQUID magnetometry - measurement uncertainties

There are three main sources of uncertainty of the magnetic properties determination:

- due to errors during the weight measurement of empty and full capsule (systematic and random)
- due to the diamagnetic contribution from the Parafilm® envelope (systematic)
- due to imperfection of the measurement of the magnetic moment of the sample by the SQUID magnetometer.

Diamagnetic contribution of the envelope was estimated based on appropriately scaled measurements of the reference Parafilm® sample and subtracted from experimental data. Thus the second contribution was approximately taken care of and further neglected.

The mass of the sample was determined to be the difference between the means of measured masses of full and empty capsule:

$$m_{sample} = m_{full} - m_{empty} \qquad (1)$$

Five mass readings were performed for each mass measurements. The magnetization of the sample was estimated using the formula:

$$M = \frac{\widetilde{m}}{m_{sample}} = \frac{\widetilde{m}}{m_{full} - m_{empty}} \qquad (2)$$

where $\widetilde{m}$ is the magnetic moment of the sample, measured by the SQUID magnetometer [1] and M is the (mass) magnetization of the sample.

Thus, concerning the uncertainties propagation principles [2] and assuming the independence of measurement errors, the measurement uncertainty of the sample magnetization can be described as:

$$s_M = \sqrt{s_{m_{full}}^2 \cdot \left(\frac{\partial M}{\partial m_{full}}\right)^2 + s_{m_{empty}}^2 \cdot \left(\frac{\partial M}{\partial m_{empty}}\right)^2 + s_{\widetilde{m}}^2 \cdot \left(\frac{\partial M}{\partial \widetilde{m}}\right)^2} \qquad (3)$$

The uncertainty of a mass measurement (of either empty or full envelope) was assumed to be the quadratic sum of random error, estimated by the Standard Deviation of Mean (SDOM, s_{stat}), and the systematic error, estimated by the Repeatability of the ultramicrobalance ($d =$ 250 ng, [3]):

$$s_m = \sqrt{s_{stat}^2 + d^2} \qquad (4)$$

The measurement uncertainty of the magnetic moment of the sample ($s_{\widetilde{m}}$) was estimated by the instrument for each measurement and further used explicitly in calculations. Finally, the calculated uncertainty of the mass magnetization can be expressed as:

$$s_M = \sqrt{M^2 \frac{2d^2 + s^2_{stat_{empty}} + s^2_{stat_{full}}}{\left(m_{full} - m_{empty}\right)^2} + s^2_{\widetilde{m}} \frac{1}{\left(m_{full} - m_{empty}\right)^2}} \quad (5)$$

which was the final definition of magnetization uncertainty used throughout this work.

Coercivity (h_c) of the sample was estimated using linear interpolation of hysteresis between two points closest to the root:

$$h_c = -\frac{M_1 h_2 - M_2 h_1}{M_2 - M_1} \quad (6)$$

where M_i and h_i are the magnetization and the magnetic field strength at i^{th} point. Accordingly, assuming the independence of uncertainties, the uncertainty of this estimation can be expressed as:

$$s_{h_c} = \sqrt{s^2_{M_1} \cdot \left(\frac{\partial h_c}{\partial M_1}\right)^2 + s^2_{M_2} \cdot \left(\frac{\partial h_c}{\partial M_2}\right)^2 + s^2_{h_1} \cdot \left(\frac{\partial h_c}{\partial h_1}\right)^2 + s^2_{h_2} \cdot \left(\frac{\partial h_c}{\partial h_2}\right)^2} \quad (7)$$

Assuming that the estimation of the magnetic field strength using the SQUID magnetometer is accurate ($s_{h_i} = 0$), one can derive the equation for the uncertainty of coercivity:

$$s_{h_c} = \sqrt{(h_1 - h_2)^2 \cdot \frac{M_1^2 s^2_{M_2} + M_2^2 s^2_{M_1}}{(M_1 - M_2)^4}} \quad (8)$$

which was the final definition used throughout this work.

1.2. Temperature-dependent measurements of magnetization

ZFC/FH/FC experiment was performed in order to determine the blocking temperature of samples. It consisted of three phases:

1. Cooling the sample from 315.0 K down to 2.0 K without external magnetic field (*zero-field cooling*, ZFC).
2. Heating the sample back up from 2.0 K to 315.0 K under 100 Oe external magnetic field (*field heating*, FH).
3. Cooling the sample down to 2.0 K under 100 Oe external magnetic field (*field cooling*, FC).

2. Results and discussion

2.1 Preferential adsorption of MES on the AgNPs within Fe_3O_4/Ag@MES@SiO_2 visualized with STEM-EDX

Apart from observing the distribution of main elements that build the nanocomposite: Fe, Ag, and Si, STEM-EDX mapping lets us indirectly confirm the chemical functionalization of the nanocomposite. As seen in Figure S1, the distribution of the signal due to the sulfur atoms clearly overlapped with the regions occupied by the nanocomposite (compare Figure S1A and B). The highest intensity of the S signal was consistently spotted where the Ag component of the composite was present. Since MES molecules are the sole source of S atoms in the material, this result proves that the adsorption of the SERS tag was successful. At the same time, no sulfur was detected in the areas outside of the composite which firstly validates the result as real and not an effect of noise and secondly shows that the sample is well-purified from non-adsorbed chemicals.

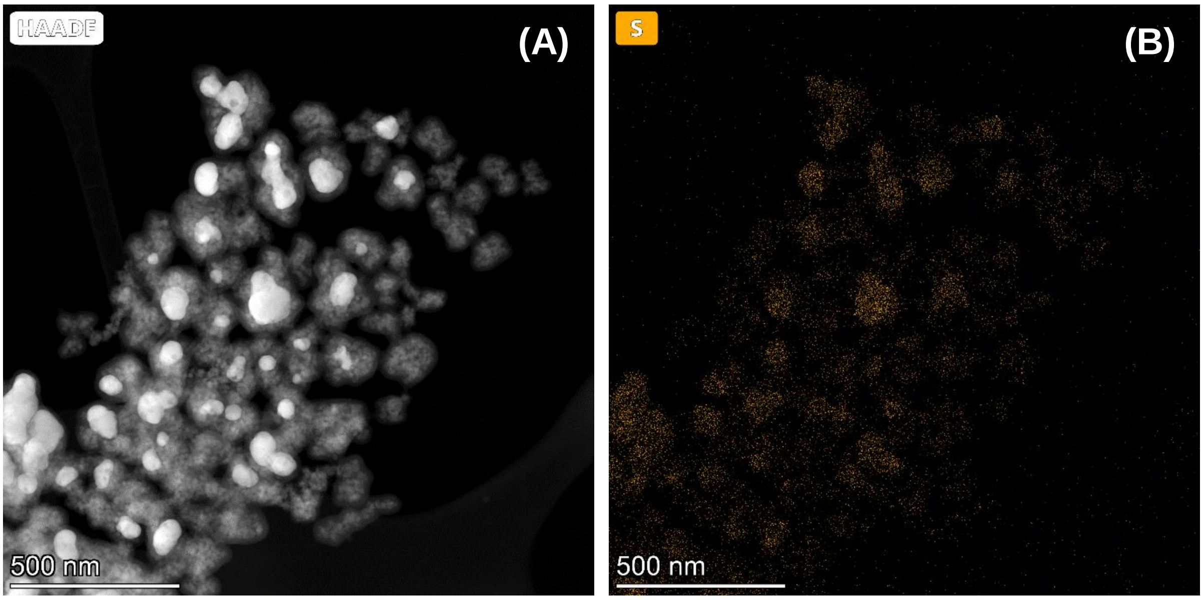

Figure S1

(A) STEM-HAADF image (also shown in Figure 3A in the main manuscript) and (B) corresponding STEM-EDX elemental mapping image showing the distribution of sulfur.

2.2 STEM-HAADF and STEM-EDX images of an individual cluster of Fe_3O_4/Ag@MES@SiO_2

STEM-HAADF combined with STEM-EDX imaging clearly resolved the atomic distribution within the single cluster of Fe_3O_4/Ag@MES@SiO_2 (Figure S2). The large silver nanoparticle is surrounded by smaller in size Fe-rich NPs (it is reasonable to assume they are composed of iron oxide) and all of those are surrounded by Si-enriched layer, as can be easily noticed in Figure S2 B-D.

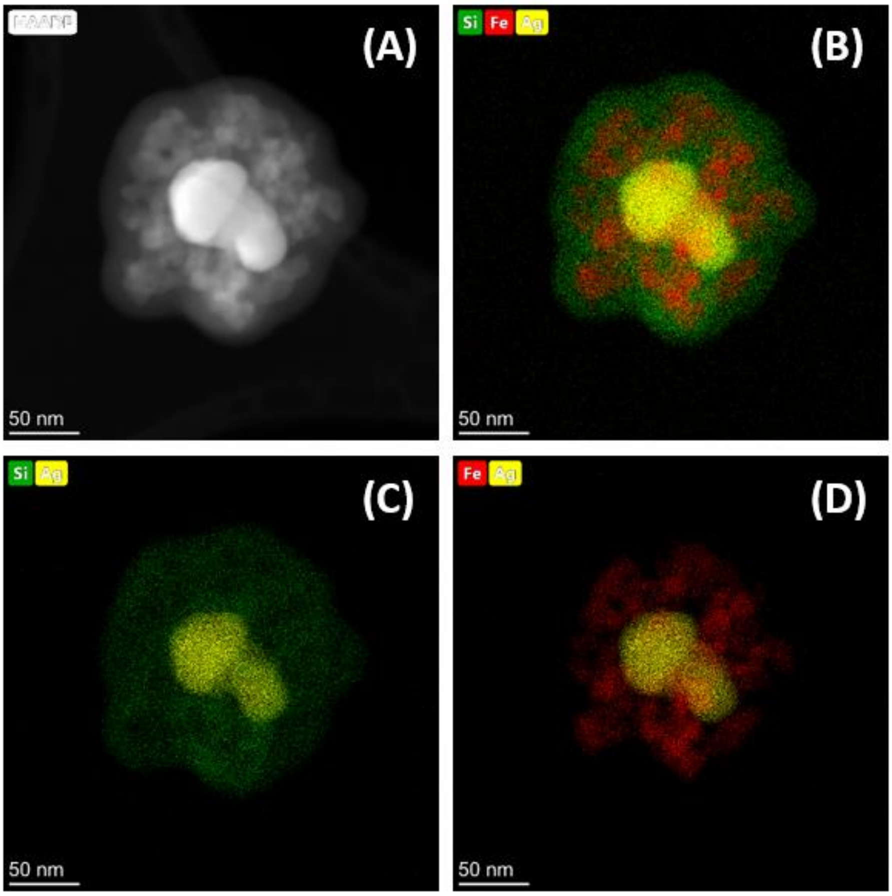

Figure S2

Nanostructural characterization and chemical composition of small cluster of Fe_3O_4/Ag@MES@SiO_2 nanocomposite:

(A) STEM-HAADF and corresponding (B)-(D) STEM-EDX elemental mapping images showing the distribution of respectively overlay of Si+Fe+Ag, Si+Ag and Fe+Ag representative for the area scanned to acquire image presented in part (A).

2.3 Zero-field cooling/field heating/field cooling (ZFC/FH/FC) experiment and evidence for population of superparamagnetic Fe_3O_4 NPs

Zero-field cooling/field heating/field cooling (ZFC/FH/FC) experiment (see Figure S3) was performed to determine the blocking temperature for the neat and further modified Fe_3O_4 nanomaterial. ZFC and FC curves were normalized for each sample by dividing the values of magnetization measured at a particular temperature by the magnetization obtained at 315 K (which was the upper bound of the temperature sweep). As evidenced in Figure S3, the shapes obtained for the ZFC/FC curves of Fe_3O_4/Ag@MES and Fe_3O_4/Ag@MES@SiO_2 samples are nearly identical, further confirming the unchanged magnetic character of the Fe_3O_4 core under silanization conditions. There is some small change between the Fe_3O_4 sample and other samples, as the magnetization increase is steeper for the modified samples. This can be qualitatively explained by the probable smaller mean diameter of Fe_3O_4 nanoparticles in the modified samples as opposed to the neat sample. All examined samples showed irreversible behavior (i.e. separated ZFC and FC curves) up to 315.0 K, indicating that there is a considerable population of magnetic nanoparticles in the sample that are blocked even at room temperature. No distinct maximum was found in any of the measured FH curves. Both these features rule out the existence of only very small and monodisperse, single-domain superparamagnetic nanoparticles of Fe_3O_4 in the samples. This result is consistent with the particle size required in case of Fe_3O_4 for the emergence of superparamagnetic properties, commonly estimated to be below 20 nm or even less [4,5]. Please bear in mind that here synthesized NPs barely approach this threshold value, having an average size around 20 nm. The consistent behavior of the blocking temperature across the synthetic pathway (see Table S1) implies that the magnetic character of the Fe_3O_4 core was unchanged upon the formation of the composite.

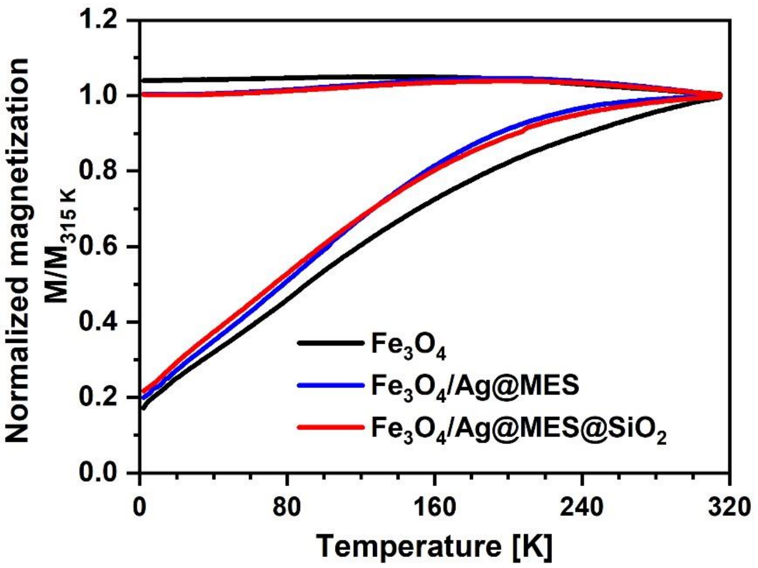

Figure S3

Normalized results of the ZFC/FC (*zero-field heating/field cooling*) experiment for the bare Fe_3O_4, Fe_3O_4/Ag@MES and Fe_3O_4/Ag@MES nanoparticles.

Strict definition of superparamagnetism requires two conditions to be met [6]:

- magnetization of the material as a function of the external magnetic field must not have any coercivity at a given temperature
- magnetization defined as a function of the external magnetic field divided by the temperature must be independent of the temperature (i.e. magnetization at a given magnetic field should be inversely proportional to the temperature).

It is worth noting, however, that many contemporary papers (especially outside solid state physics, e.g. in materials science or chemistry) disregard the second condition and seem to only be interested in the first condition [7–9], which is undeniably met for the here examined Fe_3O_4 nanomaterial (Figure 4 in the manuscript). Some works also include the analysis of ZFC/FC curves in order to determine the blocking temperature of nanoparticles [10–12] above which the material is thought to be superparamagnetic. This cannot be easily done for our case, as the blocking temperature is not defined well for our material, which is evidenced by the ZFC/FC experiments where ZFC and FC merge only at the upper bound of the experiment temperature range. (Figure S3).

At the same time, second condition cannot be reliably evaluated, as at 2 K magnetic properties show obvious hysteretic behavior (data not shown), ensuring that superparamagnetism can be ruled out at that temperature on the basis of the first condition. This means that, as worded by Hurd [6], superparamagnetism is already *destroyed* at that temperature. Full examination of the

second condition was not performed, as the magnetization of samples was measured only at 2.0 K and 300.0 K (Table 2 in the manuscript and Table S1).

In conclusion, basing solely on the first condition of superparamagnetism, which is not uncommon in the literature, it could be said that our nanomaterials are superparamagnetic. However, taking the ZFC/FC experiments into consideration, as the blocking temperature of the full samples cannot be defined in the experimental temperature region, at least it can be said that a certain population of nanoparticles in the samples is superparamagnetic, resulting in zero coercivity (Figure 4). Remaining nanoparticles are still ferromagnetic at 300.0 K, leading to the divergence of ZFC and FC curves.

Table S1 Magnetic properties and estimated mass composition of the Fe_3O_4/Ag@MES@SiO_2 and intermediate products based on SQUID magnetometry measurement

Sample	Fe_3O_4	Fe_3O_4/Ag	Fe_3O_4/Ag@MES	Fe_3O_4/Ag@MES@SiO_2
Mass of the sample [μg]	1210.2 ± 2.3	383.7 ± 4.4	90.06 ± 0.56	78.14 ± 0.51
Saturation magnetization – 2 K [emu/g]	87.82 ± 0.17	38.47 ± 0.44	36.94 ± 0.27	29.46 ± 0.19
Coercivity – 2 K [Oe]	207.9 ± 1.1	236.32 ± 0.49	239.00 ± 0.44	236.0 ± 1.2
Saturation magnetization – 300 K, highest observed magnetization method	77.20 ± 0.15	33.56 ± 0.39	32.64 ± 0.24	26.25 ± 0.17
Saturation magnetization – 300 K, Langevin function fit	72.78 ± 0.85	31.83 ± 0.37	31.24 ± 0.32	25.36 ± 0.23
Mean magnetic moment of a single particle [μ_B]	24200 ± 1000	25200 ± 1100	25160 ± 960	24640 ± 830
Coercivity – 300 K [Oe]	$10 \cong 0$	16.95 ± 0.30	17.73 ± 0.26	11.86 ± 0.20
Blocking temperature [K]	-	-	$\gg 315$	$\gg 315$
Mass concentration [%] of the components	$Fe_3O_4 - 100$	$Fe_3O_4 - 43.47 \pm 0.51$<br>$Ag - 56.53 \pm$ 0,51	$Fe_3O_4 - 42.28 \pm 0.32$<br>$Ag - 55.0 \pm 1.2$<br>$MES - 2.7 \pm 1.2$	$Fe_3O_4 - 34.00 \pm 0.23$<br>$Ag - 44.22 \pm 0.96$<br>$MES - 2.2 \pm 1.1$<br>$SiO_2 - 19.6 \pm 1.5$

2.4 Raman characterization of Fe_3O_4/Ag@MES using 532 nm laser

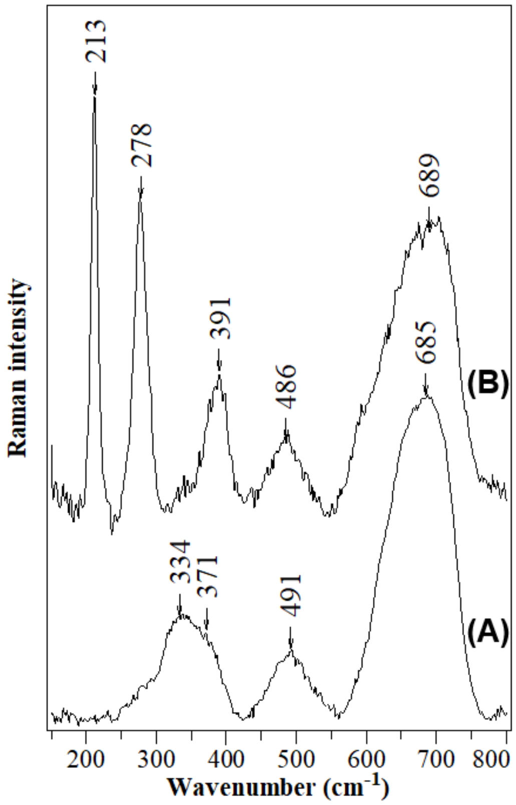

Figure S4

Raman spectra excited with 532 nm wavelength (2x60 s acquisition time for each spectrum, laser power on the sample around 3 mW) of Fe_3O_4/Ag@MES: (A) initial and (B) upon prolonged irradiation with laser. The spectra were baselined and intensity shifted for the clarity of presentation.

2.5 Characterization of the intermediate products during the optimization of the synthesis of nanocomposites

Initial synthesis based on the protocol by Kim et al. [13] and preliminarily adjusted to our system (Chapter *Decoration of magnetite with AgNPs* of the main article) clearly showed a few obstacles to overcome, namely high total content of the AgNPs in the sample, excessive amount of the large silver nanostructures, and their high size polydispersity. Too high contribution of diamagnetic silver was expected to have an adverse impact on the magnetic properties of the nanocomposite. Dimensions of the nanoparticles are on the other hand fundamental for their SERS performance[14]. Larger NPs are expected to provide higher electromagnetic enhancement [15], however their increased size leads as well to predominance of light scattering over absorption by the NPs [16], which in general should reduce the overall SERS intensity. Certainly, enhancement by electromagnetic field is also strongly dependent on the excitation wavelength. It was initially predicted theoretically that maximum electric field enhancement on the surface of silver spheres should appear for the particles with the 25 nm diameter [17]. Further increase in size should result in reduced dipolar plasmon resonance due to combination of dynamic depolarization and radiation-damping effects. More recent simulations [15] showed that silver nanoparticles exhibit high electric field enhancement when their size approaches 70-100 nm size range for the 632.8 nm excitation wavelength (identical with that used here for SERS experiment). Indeed, higher SERS signal and enhancement factor were observed experimentally for the 632.8 nm laser source while increasing the size of the spherical silver nanoparticles up to 65 nm (in diameter) [18,19]. It was further confirmed by finite simulations of electromagnetic enhancement factor, however the key role of particle spacing was also stressed [19].

Based on the above considerations, it can be safely assumed that few tens of nm in size silver nanospheres (larger than 25 nm) are considered to provide the best SERS activity for here applied excitation wavelength (632.8 nm). Very large AgNPs are undesirable due to high scattering efficiency. On the other hand, broad size distribution of the nanostructures is also unwelcome, as it could readily result in a non-uniform surface enhancement across the sample. Consequently, we investigated the effect of the relative quantitative relation between Ag^+ ion and reducing agent concentrations, as well as amount of Fe_3O_4 NPs on the morphology of the fabricated Fe_3O_4/Ag nanocomposite.

To achieve that, systematic changes in the amount of reagents (amount of Fe_3O_4 NPs, concentration of silver nitrate and molar ratio of Ag^+ to *n*-$ButNH_2$) were applied to the

procedure. Quality control of the products derived from those modifications to the experimental protocol was carried out by means of TEM microscopy (here, AgNPs appear as darker features in the image). Figure S5 presents TEM images (all at the same magnification) of the Fe_3O_4/Ag nanocomposite prepared using $AgNO_3$ solution with concentration reduced to 4 mM (Figure S5A-C) or 2 mM (Figure S5D). Quantitative data concerning the average Ag nanoparticle size and its absolute and relative standard deviation determined for this set of results can be found in Table S2. Respective size histograms of AgNPs are also compared in Figure S6. Images in Figure S5A and B were taken for nanocomposites synthesized with the same amount of magnetite NPs (2 mg/5 ml) and identical (4 mM) concentration of $AgNO_3$, while the Ag^+ to *n*-ButNH$_2$ molar ratio was changed from 1:1 (part A) to 5:1 (part B). Increasing ratio of the metal ions with respect to *n*-ButNH$_2$ indeed resulted in a higher number of the silver nucleation centers, at the same time barely affecting the size distribution. Being more specific, the size of the AgNPs was reduced almost twice (from 60.8 nm to 36.1 nm) when molar ratio of silver ions to reducing agent was increased from 1:1 to 5:1, while the relative standard deviation of (RSD) of size distribution (defined as the ratio between the standard deviation and the average size) remained at the level of around 33%. Such a small size (around 35 nm) seems to be unfavorable from the point of view of SERS activity of AgNPs.

To compare the effect of reduced total amount of silver ions introduced into the reaction mixture, the TEM images acquired for 4 mM (Figure S5C) and 2 mM $AgNO_3$ (Figure S5D) for the fixed amount of Fe_3O_4 (1 mg/mL) and Ag^+ to *n*-ButNH$_2$ molar ratio (5:1) should be analyzed. It can be clearly seen that the use of 2 mM $AgNO_3$ concentration is already too low, as the AgNPs are hardly formed at all (see Figure S5D). Moreover, they become too small to exhibit SERS activity (Feret diameter below 30 nm). Interestingly, the results discussed so far, presented in Figure S5 and S6) show comparable values of RSD of size (Table S2), which proves the general reproducibility of here established synthesis protocol.

On the other hand, examining TEM images showing exclusively the influence of magnetite NPs amount (compare Figure S5B and C) demonstrates that reducing the magnetite NPs amount from 2 mg/5 ml to 1 mg/5 ml results in heavily increased content of the silver nanostructures (compare Figure S5B and C). This regrettably leads to the formation of unwanted, randomly distributed hot spots (Figure S5C) and potential risk of strongly reduced magnetization of such nanomaterial. For these reasons we fixed the magnetite NPs amount and Ag^+ ions to *n*-ButNH$_2$ molar ratio to 2 mg/5 mL and 1:1, respectively, and further studied the effect of silver ions concentration.

In Figure S7 effect of systematic changes of $AgNO_3$ concentration on structural features of Fe_3O_4/Ag nanocomposites obtained with use of 2 mg/5 mL of magnetite NPs and 1:1 Ag^+ to *n*-$ButNH_2$ molar ratio can be followed, with aid of TEM analysis. TEM images at two different magnifications are compared in left and right column of this figure (for the same sample in a given row). Again, the results of a detailed size distribution analysis are summarized in Table S2, while the comparison of size histograms is presented in Figure S8. However, already at the very first glance on Figure S8, it can be seen that increased concentration of $AgNO_3$ results in formation of larger Ag NP – reaching the value as high as 79.8±38.9 nm, as evidenced by TEM images in Figure S7A and A'. However, the two discussed images show clearly that the AgNPs are very tightly packed for 16 mM, which is expected to give rise to substantial differences in the values of local SERS intensity. Additional disadvantage is that estimated value of RSD increased to nearly 50% for the sample prepared with the highest investigated concentration of $AgNO_3$ (16 mM), comparing to other protocols for this step of synthesis (Table S2)

Synthesized AgNPs of considerably smaller size (below 61 nm, see Table S2 for the details) and with a visibly reduced total contribution of silver content in the Fe_3O_4/Ag nanocomposite can be found for 4 mM and lower values of concentration of $AgNO_3$ (see TEM images in Figure S7C and C', together with D and D'). It is also worth emphasizing that collected TEM images reveal considerable disadvantageous agglomeration of the magnetic component of the nanocomposite, particularly noticeable for the two latter experimental conditions.

Table S2 Effect of the experimental protocol on the average size and (R)SD of AgNPs

**Content of Fe_3O_4 NPs**	**Molar ratio of $[Ag^+]/[n\text{-}ButNH_2]$**	**Concentration of $AgNO_3$ [mM]**	**Particle size* Gauss fit [nm]**	**Particle size Mean value [nm]**	**SD**	**RSD [%]**
1 mg/5 mL	5:1	2	25.6	29.1	±10.5	36,1
		4	42.4	45.8	±15.3	33,4
2 mg/5 mL			33.2	36.1	±12.0	33,2
2 mg/5 mL	1:1	2	46.8	49.4	±16.4	33,2
		4	57.0	60.8	±20.1	33,1
		8	63.7	72.5	±32.7	45,1
		16	67.7	79.8	±38.9	48,7

*defined as Feret's diameter

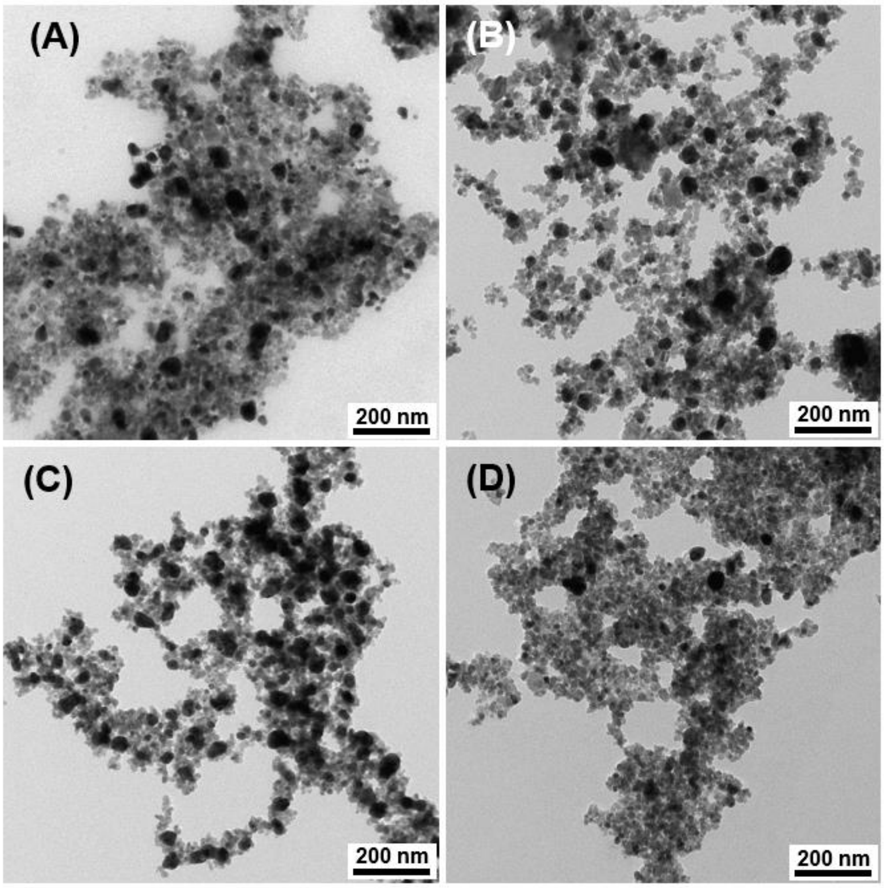

Figure S5

TEM images of Fe_3O_4/Ag nanocomposite synthesized with 2 mg/5 mL of magnetite NPs, 4 mM $AgNO_3$ and 1:1 (A) or 5:1 (B) Ag^+ to *n*-ButNH_2 ratio compared with the Fe_3O_4/Ag nanocomposite obtained with 1 mg/5 mL of magnetite NPs, 5:1 Ag^+ to *n*-ButNH_2 ratio and 4 mM (C) or 2 mM (D) $AgNO_3$.

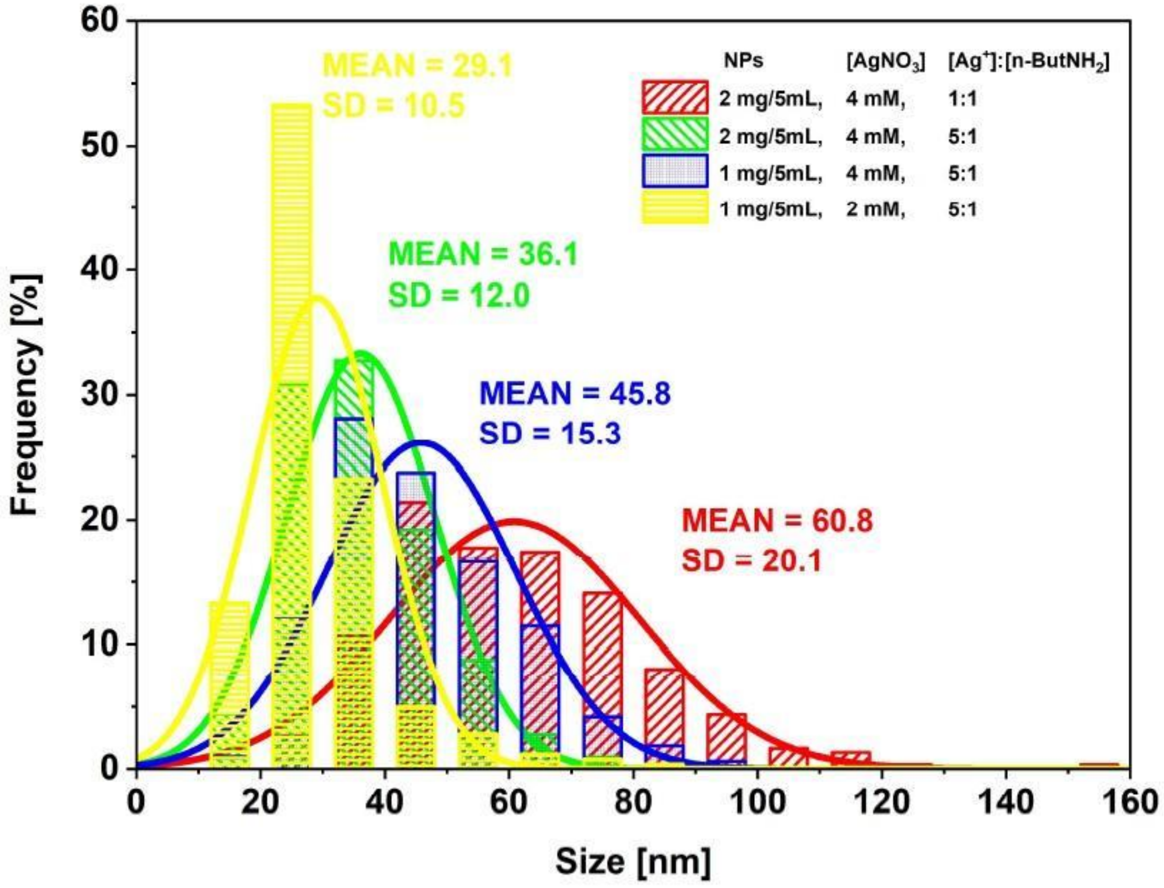

Figure S6 Size distribution histograms of AgNPs for the Fe_3O_4 nanocomposite synthesized with 2 mg/5 mL of magnetite NPs, 4 mM $AgNO_3$ and 1:1 (red) or 5:1 (green) Ag^+ to *n*-$ButNH_2$ ratio compared with the Fe_3O_4/Ag nanocomposite obtained with 1 mg/5 mL of magnetite NPs, 5:1 Ag^+ to *n*-$ButNH_2$ ratio and 4 mM (blue) or 2 mM (yellow) $AgNO_3$.

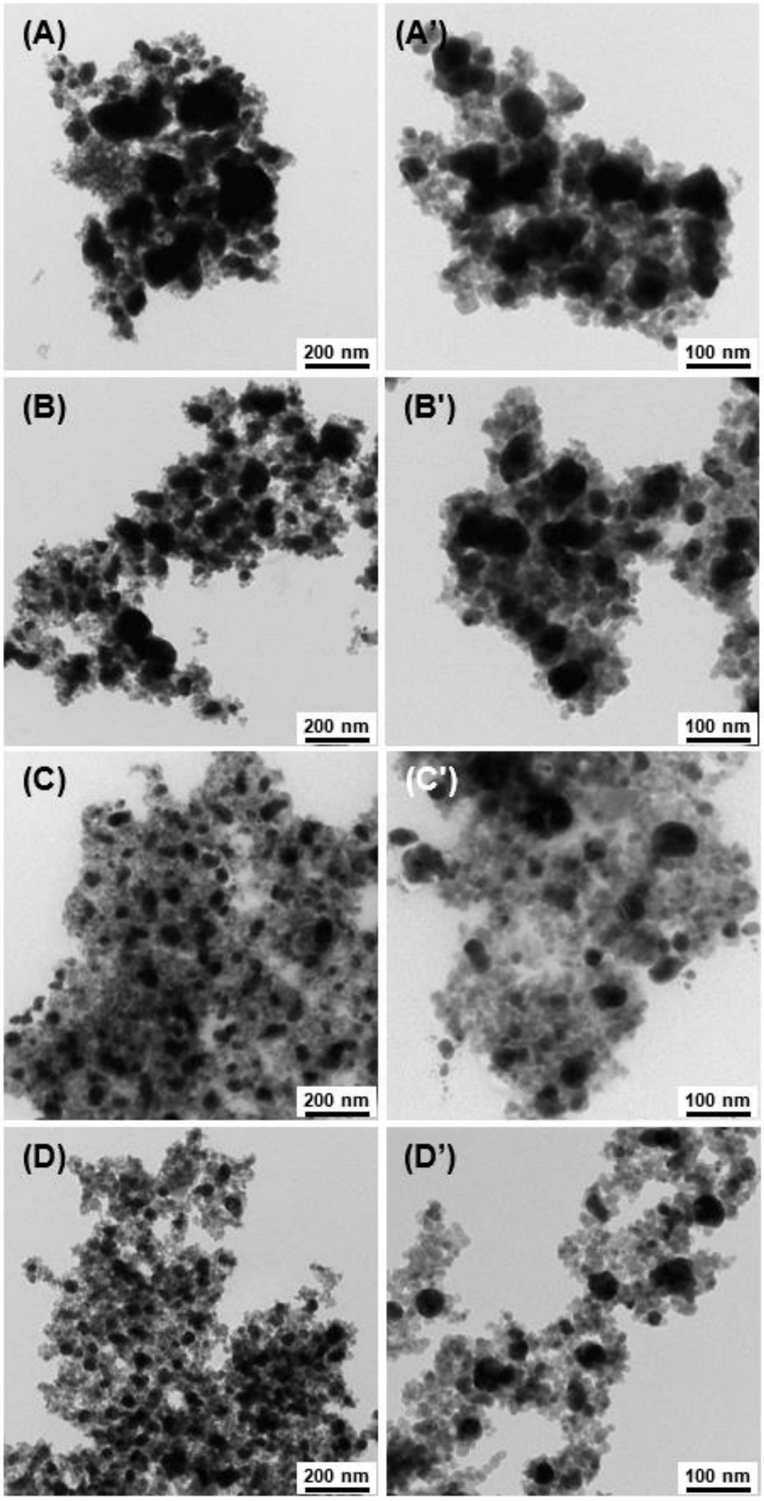

Figure S7 TEM images of Fe_3O_4/Ag nanocomposite synthesized with 2 mg/5 mL of magnetite NPs, 1:1 Ag^+ to *n*-ButNH_2 molar ratio and varying $AgNO_3$ concentration: 16 mM (A) and A'), 8 mM (B and B'), 4 mM (C and C') and 2 mM (D and D').

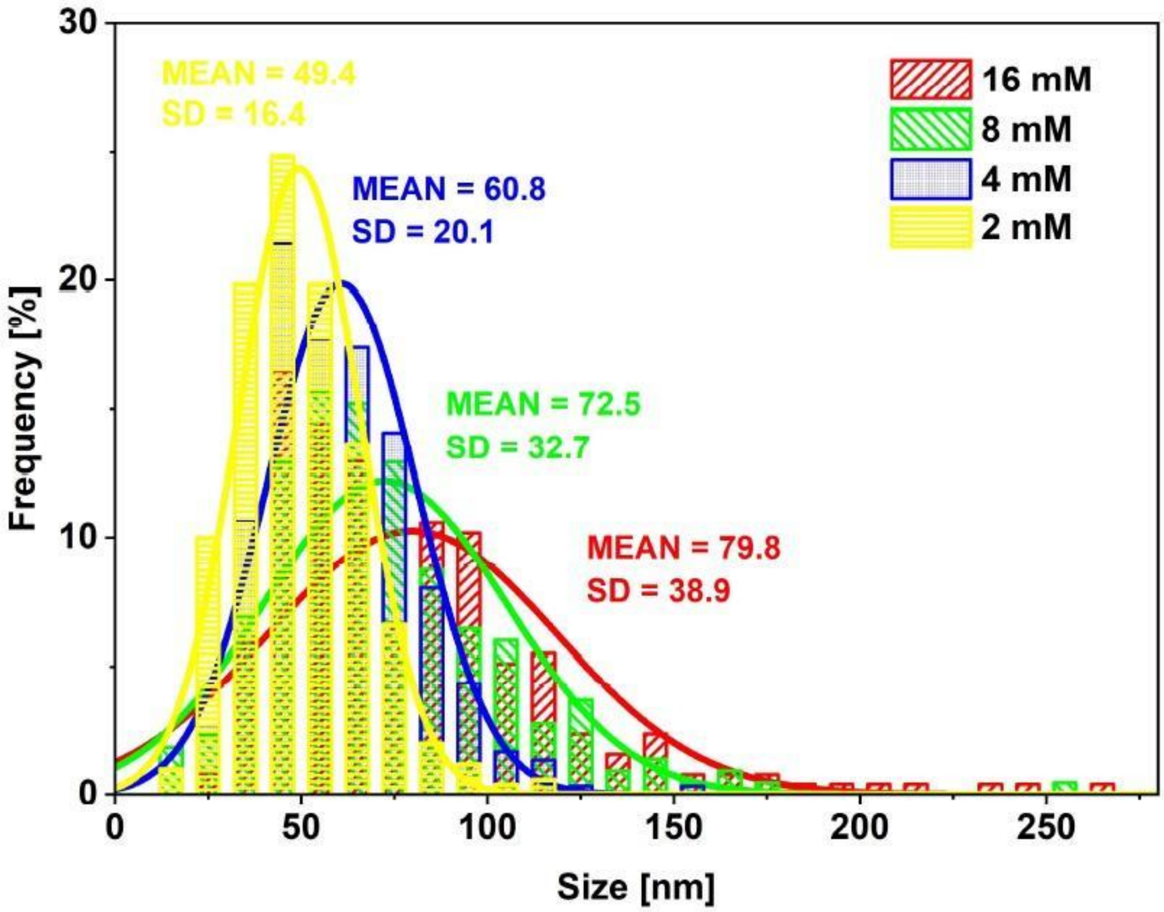

Figure S8 Size distribution histograms of AgNPs for the Fe_3O_4/Ag nanocomposite synthesized with 2 mg/5 mL of magnetite NPs, 1:1 Ag^+ to *n*-ButNH_2 molar ratio and varying $AgNO_3$ concentration: 16 mM (red), 8 mM (green), 4 mM (blue) and 2 mM (yellow).

Table S3 Values of the mean SERS intensity (with SD and RSD) for the composites obtained with 2 mg/5 mL of Fe_3O_4 NPs, 1:1 molar ratio of molar ratio of $[Ag^+]/[n\text{-}ButNH_2]$ and varying concentration of silver ions

Concentration of $AgNO_3$ [mM]	Average SERS intensity for given area [cnt/s]	SD [cnt/s]	RSD [%]
2	40*	36	90
	42	23	55
4	59	24	41
	60	23	38
8	454	144	32
	459	145	32
	466	112	24
16	221	70	32
	282	123	44

*Typically 25 spots localized across 100 μm x 150 μm rectangular area were analyzed. The intensity values were normalized, according to the procedure

2.6 Impact of method of purification on the structural and SERS properties of the final product

Impact of various methods of purification (rinsing the final product consecutively with water, ethanol, and water: "WEW" or three times with water only: "3xW") on morphology and SERS performance of the final nanocomposite was studied. The WEW order was initially applied, as this protocol was successfully employed to purify the Fe_3O_4/Ag and Fe_3O_4/Ag@MES intermediate products, based on the protocol used by Kim et al. [13] for equivalent silanization procedure. We decided to eliminate ethanol from the washing procedure, as small silica particles prepared using the Stöber method show stronger swelling behavior in ethanol than in water [20]. The magnet-assisted collection of the here examined material at each step continued up to 2 hours, which is much shorter than in reference [20]. Still, we expected that thinner silica shells will be grown when replacing ethanol with water. Therefore, we also chose to check the influence of rinsing the final product with water only (3xW). STEM images are presented in Figure S9), discussion can be found in Chapter *Influence of silica coating and method of purification* of the main article.

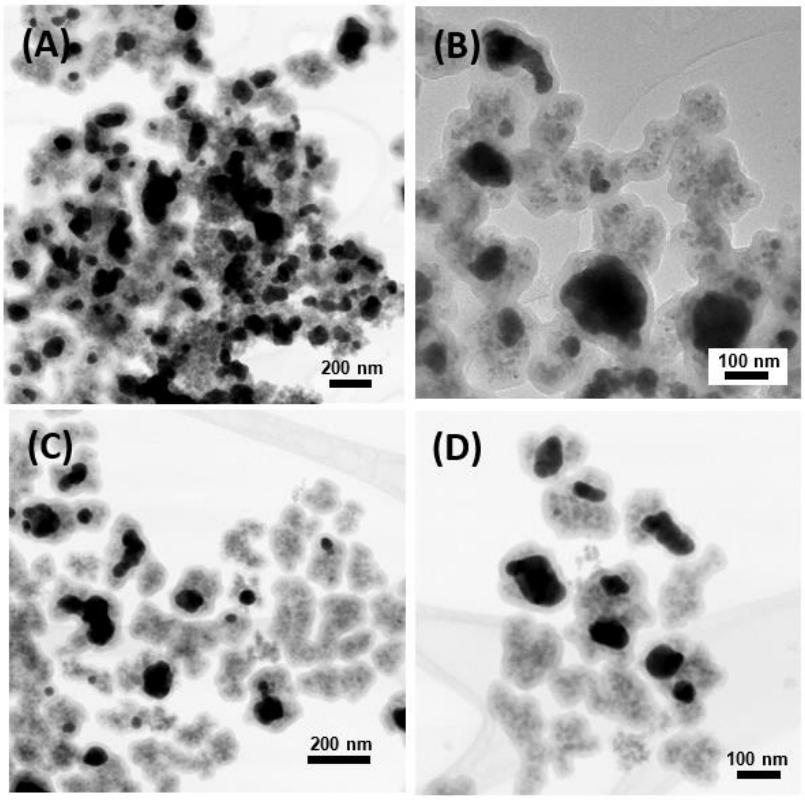

Figure S9

STEM images of Fe_3O_4/Ag/MES/SiO_2 nanocomposite synthesized according to optimized protocol and purified: (A) and (B) with water-ethanol-water (WEW); and (C) and (D) with water only (3xW).

Comparison of SERS response for the Fe_3O_4/Ag@MES@SiO_2 nanocomposite purified by these two methods, presented in Figure S10A, shows that the use of water (3xW) is advantageous. Firstly, MES layer was more prone to thermal decomposition when ethanol was used, as evidenced by the clearly captured spectral fluctuations, characteristic of amorphous carbon] [21] (see the bottom spectrum in Figure S10A as a representative one). Secondly, probably this thermal degradation of the MES monolayer resulted sometimes in appearance of the Raman bands typical of magnetite (compare middle spectrum in Figure S10A with Figure S4A). However, the manifestation of iron oxide Raman signature can also be related to the presence of pure magnetite nanoparticles coated with silica, as observed in Figure S9A and B. The degradation of the Raman tag itself can be associated with the appearance of the tightly-packed agglomerates of the Ag component, in Figure S9A, leading to too high SERS activity of the such formed hot spots and decomposition of MES layer.

Raman maps showing the distribution of the intensity of the marker bands of amorphous carbon (broad feature in the range 1500-1600 cm^{-1}) and iron oxide (integrated intensity in the range 650-750 cm^{-1}) across the two areas of the WEW purified sample are shown respectively in Figure S10B and Figure S10C. On the other hand, the undisturbed SERS spectrum indicative of MES molecules was the prevailing one when mapping the Fe_3O_4/Ag@MES@SiO_2 sample washed with water only (3xW; see the top spectrum in Figure S10 as the representative one).

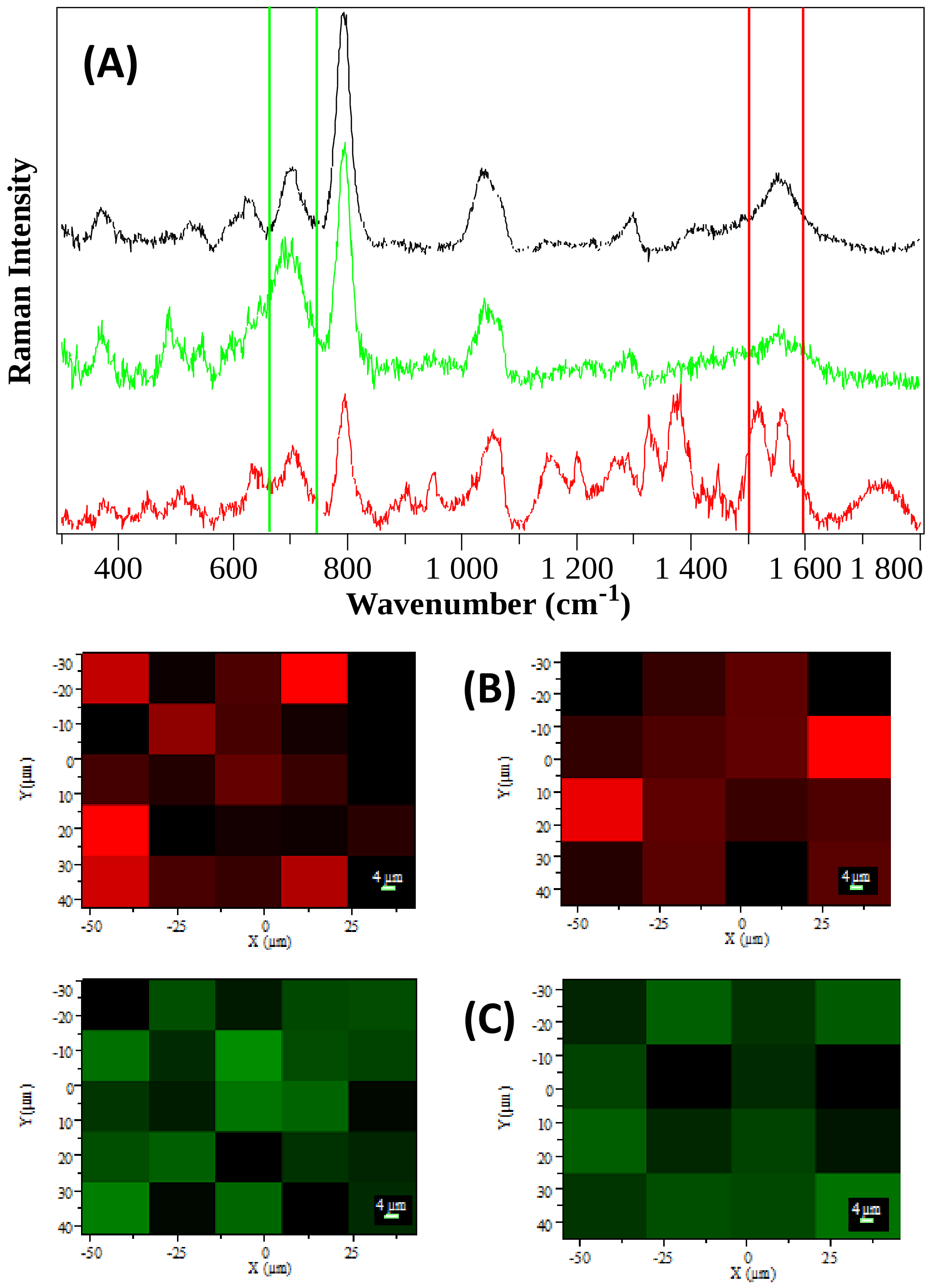

Figure S10

(A) Representative SERS spectra excited with 632.8 nm wavelength (3x10 s acquisition time for each spectrum, laser power on the sample around 0.75 mW)of Fe_3O_4/Ag@MES@SiO_2 for the samples purified with water only (3xW) – top one; and water-ethanol-water (WEW) – middle and bottom one. The spectra were baselined and intensity shifted for the clarity of presentation.

Raman maps plotting integrated intensity of the marker band of (B) amorphous carbon (1500-1600 cm^{-1}) and (C) magnetite (650-750 cm^{-1}) in the two scanned areas of the WEW sample. The more intense the color in the map, the higher the value of the integral intensity. The color coding is consistent for the (B) and (C) maps and markers integral limits in part (A).

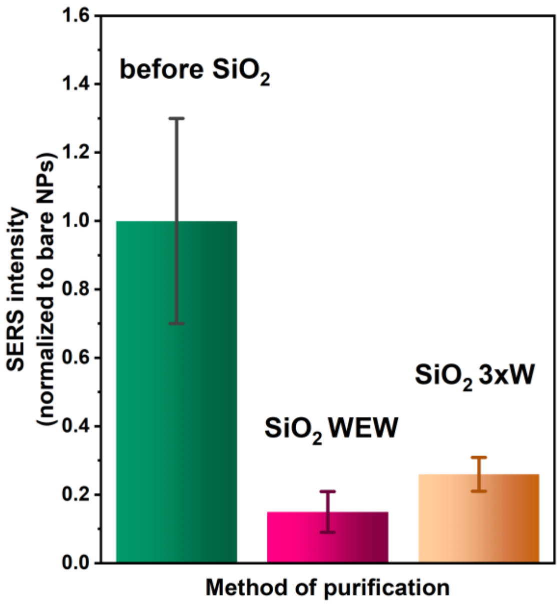

Figure S11

Effect of SiO_2 coating and method of purification on average intensity of MES SERS band around 790 cm^{-1} (with error bars showing the RSD values). The results are normalized to the SERS intensity measured for bare Fe_3O_4/Ag. The values of RSD were also recalculated according to this procedure. Typically 16-25 spots localized across 80 μm x 100-120 μm rectangular area were analysed in each case.

2.7 Effect of magnetic field on assembly of bare and silica-coated nanocomposite

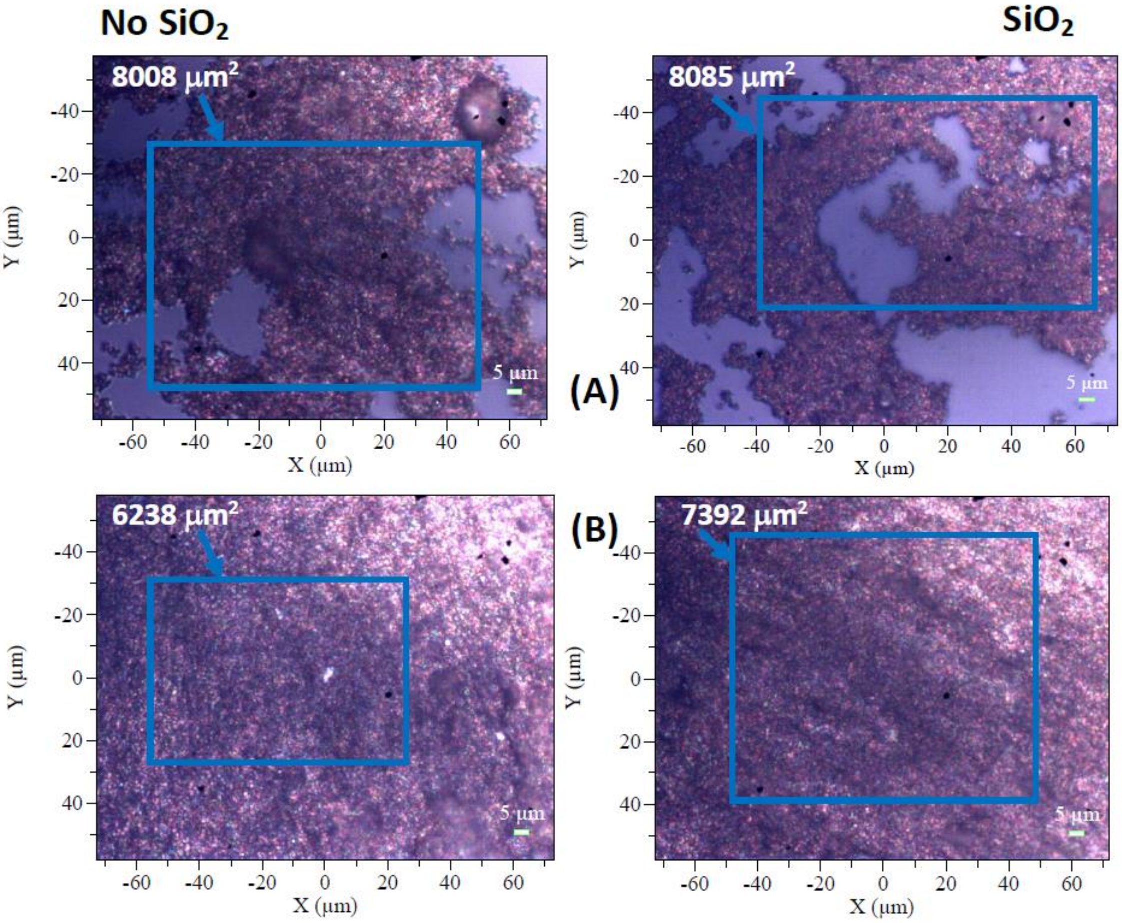

Figure S12

Optical images of the Fe_3O_4/Ag@MES (left column) and Fe_3O_4/Ag@MES@SiO_2 (right column) self—assembled (A) and magnet-assembled (B) onto glass substrate. The blue rectangle marks the area analyzed with SERS spectroscopy. The exact value of the scanned areas for each sample is given in the legend, with an arrow pointing to scanned region.

2.8 Temporal evolution of UV-Vis spectra upon mixing with yeast for bare nanocomposite

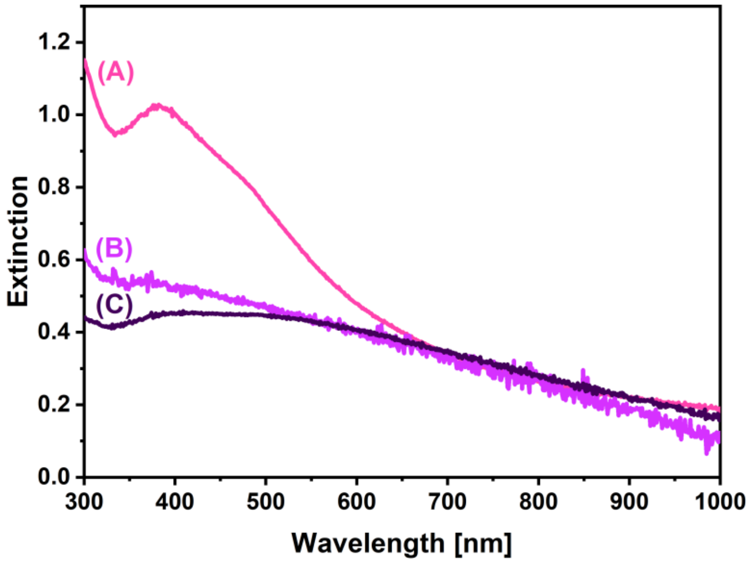

Figure S13

Changes in the extinction spectra upon addition of yeast cells to suspension of Fe_3O_4/Ag@MES nanocomposite. Spectra collected (A) before and after (B) 1 hour and (C) 48 hours of contact with yeast.

3. References

[1] M. McElfresh, Fundamentals of Magnetism and Magnetic Measurements: Featuring Quantum Design's Magnetic Property Measurement System, Quantum Design, 1994.

[2] J.R. Taylor, An introduction to error analysis: The study of uncertainties in physical measurements, 2nd ed., University Science Books, Sausalito, Caifornia, 1997.

[3] Sartorius ME and SE Series: Operation Instructions, (n.d.).

[4] C.T. Yavuz, J.T. Mayo, W.W. Yu, A. Prakash, J.C. Falkner, S. Yean, L. Cong, H.J. Shipley, A. Kan, M. Tomson, D. Natelson, V.L. Colvin, Low-field magnetic separation of monodisperse Fe3O4 nanocrystals, Science (80-.). 314 (2006) 964–967. doi:10.1126/science.1131475.

[5] D. Huber, Synthesis, Properties, and Applications of Iron Nanoparticles, Small. 1 (2005) 482–501. doi:10.1002/smll.200500006.

[6] C.M. Hurd, Varieties of magnetic order in solids, Contemp. Phys. 23 (1982) 469–493. doi:10.1080/00107518208237096.

[7] Ø. Olsvik, T. Popovic, E. Skjerve, K.S. Cudjoe, E. Hornes, J. Ugelstad, M. Uhlén, Magnetic separation techniques in diagnostic microbiology, Clin. Microbiol. Rev. 7 (1994) 43–54. doi:10.1128/CMR.7.1.43.

[8] Y.X.J. Wang, S.M. Hussain, G.P. Krestin, Superparamagnetic iron oxide contrast agents: Physicochemical characteristics and applications in MR imaging, Eur. Radiol. 11 (2001) 2319–2331. doi:10.1007/s003300100908.

[9] T. Neuberger, B. Schöpf, H. Hofmann, M. Hofmann, B. Von Rechenberg, Superparamagnetic nanoparticles for biomedical applications: Possibilities and limitations of a new drug delivery system, in: J. Magn. Magn. Mater., North-Holland, 2005: pp. 483–496. doi:10.1016/j.jmmm.2005.01.064.

[10] R.A. Bini, R.F.C. Marques, F.J. Santos, J.A. Chaker, M. Jafelicci, Synthesis and functionalization of magnetite nanoparticles with different amino-functional alkoxysilanes, J. Magn. Magn. Mater. 324 (2012) 534–539. doi:10.1016/j.jmmm.2011.08.035.

[11] K. Kijewska, A. Jarzebińska, J. Kowalska, J. Jemielity, D. Kepińska, J. Szczytko, M. Pisarek, K. Wiktorska, J. Stolarski, P. Krysiński, A. Twardowski, M. Mazur, Magnetic-nanoparticle-decorated polypyrrole microvessels: Toward encapsulation of mRNA cap analogues, Biomacromolecules. 14 (2013) 1867–1876. doi:10.1021/bm400250g.

[12] M. Bartel, B. Wysocka, P. Krug, D. Kępińska, K. Kijewska, G.J. Blanchard, K. Kaczyńska, K. Lubelska, K. Wiktorska, P. Głowala, M. Wilczek, M. Pisarek, J. Szczytko, A. Twardowski, M. Mazur, Magnetic polymer microcapsules loaded with Nile Red fluorescent dye, Spectrochim. Acta - Part A Mol. Biomol. Spectrosc. 195 (2018) 148–156. doi:10.1016/j.saa.2018.01.056.

[13] K. Kim, J.Y. Choi, H.B. Lee, K.S. Shin, Silanization of Ag-deposited magnetite particles: An efficient route to fabricate magnetic nanoparticle-based Raman barcode materials, ACS Appl. Mater. Interfaces. 2 (2010) 1872–1878. doi:10.1021/am1002074.

[14] K.L. Kelly, E. Coronado, L.L. Zhao, G.C. Schatz, The optical properties of metal nanoparticles: The influence of size, shape, and dielectric environment, J. Phys. Chem. B. 107 (2003) 668–677. doi:10.1021/jp026731y.

[15] Z. Starowicz, R. Wojnarowska-Nowak, P. Ozga, E.M. Sheregii, The tuning of the plasmon resonance of the metal nanoparticles in terms of the SERS effect, Colloid Polym. Sci. 296 (2018) 1029–1037. doi:10.1007/s00396-018-4308-9.

[16] U. Kreibig, M. Vollmer, Optical Properties of Metal Clusters, Springer Berlin Heidelberg, Berlin, Heidelberg, 1995. doi:10.1007/978-3-662-09109-8.

[17] M. Meier, A. Wokaun, Enhanced fields on large metal particles: dynamic depolarization, Opt. Lett. 8 (1983) 581. doi:10.1364/ol.8.000581.

[18] R.N. Cassar, D. Graham, I. Larmour, A.W. Wark, K. Faulds, Synthesis of size tunable monodispersed silver nanoparticles and the effect of size on SERS enhancement, Vib. Spectrosc. 71 (2014) 41–46. doi:10.1016/j.vibspec.2014.01.004.

[19] R.X. He, R. Liang, P. Peng, Y. Norman Zhou, Effect of the size of silver nanoparticles on SERS signal enhancement, J. Nanoparticle Res. 19 (2017) 1–10. doi:10.1007/s11051-017-3953-0.

[20] C.A.R. Costa, C.A.P. Leite, F. Galembeck, Size dependence of stöber silica nanoparticle microchemistry, J. Phys. Chem. B. 107 (2003) 4747–4755. doi:10.1021/jp027525t.

[21] A. Kudelski, Fluctuations of Raman spectra of hydrogenated amorphous carbon deposited on electrochemically-roughened silver, Chem. Phys. Lett. 427 (2006) 206–209. doi:10.1016/j.cplett.2006.06.043.
